# The APSES transcription factor Efg1 is a global regulator that controls morphogenesis and biofilm formation in Candida parapsilosis

**DOI:** 10.1111/mmi.12345

**Published:** 2013-08-15

**Authors:** Leona A Connolly, Alessandro Riccombeni, Zsuzsana Grózer, Linda M Holland, Denise B Lynch, David R Andes, Attila Gácser, Geraldine Butler

**Affiliations:** 1 School of Biomolecular and Biomedical Science Conway Institute, University College DublinBelfield, Dublin 4, Ireland; 2 Department of Microbiology, University of SzegedH-6726, Szeged Kozep fasor 52, Hungary; 3 Departments of Medicine and Microbiology and Immunology, University of WisconsinMadison, WI, USA

## Abstract

Efg1 (a member of the APSES family) is an important regulator of hyphal growth and of the white-to-opaque transition in *Candida albicans* and very closely related species. We show that in *Candida parapsilosis* Efg1 is a major regulator of a different morphological switch at the colony level, from a concentric to smooth morphology. The rate of switching is at least 20-fold increased in an *efg1* knockout relative to wild type. *Efg1* deletion strains also have reduced biofilm formation, attenuated virulence in an insect model, and increased sensitivity to SDS and caspofungin. Biofilm reduction is more dramatic in *in vitro* than in *in vivo* models. An Efg1 paralogue (Efh1) is restricted to *Candida* species, and does not regulate concentric-smooth phenotype switching, biofilm formation or stress response. We used ChIP-seq to identify the Efg1 regulon. A total of 931 promoter regions bound by Efg1 are highly enriched for transcription factors and regulatory proteins. Efg1 also binds to its own promoter, and negatively regulates its expression. Efg1 targets are enriched in binding sites for 93 additional transcription factors, including Ndt80. Our analysis suggests that Efg1 has an ancient role as regulator of development in fungi, and is central to several regulatory networks.

## Introduction

The transcription factor Efg1 plays a central role in regulating morphology and virulence in the major human fungal pathogen *Candida albicans*. It was first identified as an inducer of pseudohyphal growth in *Saccharomyces cerevisiae*, and subsequently shown to be required for hyphal growth in *C. albicans* (Stoldt *et al*., 1997). Strains carrying deletions of *EFG1* fail to undergo a switch from yeast to hyphal growth in response to most stimuli (Lo *et al*., 1997; Stoldt *et al*., 1997; Sudbery, 2011) although filamentation is increased under hypoxic conditions, and when embedded in solid media (Doedt *et al*., 2004; Mulhern *et al*., 2006).

Efg1 has many functions in *C. albicans*. It is required for virulence in most infection models (Lo *et al*., 1997; Chamilos *et al*., 2006; Pukkila-Worley *et al*., 2009). In addition, cells lacking *EFG1* are unable to invade reconstituted human epithelia, and endocytosis in epithelial and endothelial cells is reduced (Weide and Ernst, 1999; Dieterich *et al*., 2002).

The pleiotropic effects of Efg1 in *C. albicans* are related to its pivotal role in several networks controlling morphology and virulence. Efg1 is one of the core regulators of the white-opaque switch, an epigenetic switch between two genetically identical cell types. Efg1 is required for the maintenance of the default white phenotype (Sonneborn *et al*., 1999b; Srikantha *et al*., 2000). Switching to opaque cells is strongly correlated with mating efficiency (reviewed in Bennett and Johnson, 2005; Lohse and Johnson, 2009; Morschhauser, 2010). White and opaque cells differ in appearance; white cells are round or ellipsoid and opaque cells are elongated with ‘pimples’ on the surface (Anderson and Soll, 1987; Anderson *et al*., 1990). They also differ in behaviour; for example, white cells are preferentially phagocytosed by macrophages (Lohse and Johnson, 2008), and opaque cells do not release a chemoattractant for leukocytes (Geiger *et al*., 2004). The two cells types have significantly different transcriptional profiles (Tuch *et al*., 2010). The white-opaque switch is regulated by a network of transcription factors that includes Efg1, Wor1, Wor2 and Czf1, together with the homeodomain mating proteins a1 and alpha2 (Sonneborn *et al*., 1999b; Srikantha *et al*., 2000; Huang *et al*., 2006; Vinces *et al*., 2006; Zordan *et al*., 2006; 2007[Bibr b119],[Bibr b120]).

Efg1 is also important for the growth of *C. albicans* cells in communities, or biofilms, on plastic or cell surfaces. Cells with an *efg1* deletion form very loose biofilms on plastic consisting mostly of yeast cells (Ramage *et al*., 2002), and form defective biofilms on vaginal mucosa (Harriott *et al*., 2010). Unlike many other biofilm regulators, Efg1 is required for biofilm development under both normoxic and hypoxic (low-oxygen) conditions (Stichternoth and Ernst, 2009). Even when cells do adhere to plastic, Efg1 is required for tolerance to antifungal drugs such as azoles, amphotericin B and caspofungin (Bink *et al*., 2012).

A recent analysis by Nobile *et al*. (2012) showed that Efg1 is part of a network of six transcription factors that regulates biofilm development in *C. albicans*. The six regulators all control each other's expression; up to five factors bind to each promoter, and in addition each activates its own synthesis. The network regulates expression of at least 1000 genes, many of which are also expressed during biofilm growth. Nobile *et al*. (2012) identified several hundred intergenic regions bound by Efg1, and separately, Lassak *et al*. (2011) showed that Efg1 binds to the promoters of at least 53 genes in *C. albicans* yeast cells, including several transcription factors. Binding of Efg1 is disrupted and new binding sites appear during the yeast-to-hyphal transition. The analysis of Nobile *et al*. (2012) suggests that the biofilm regulatory network has evolved relatively recently, and is likely to be restricted to *C. albicans*.

Among the species in the *Candida* clade [or CTG clade, species in which CTG is translated as serine rather than leucine (Santos and Tuite, 1995)], only *C. albicans* and *C. dubliniensis* form hyphae, and white-opaque switching appears to be restricted to these species plus their close relative *Candida tropicalis* (Lohse and Johnson, 2009; Morschhauser, 2010; Porman *et al*., 2011; Xie *et al*., 2012). However, Efg1 is a member of the APSES family of proteins that contain a conserved basic helix–loop–helix domain (Stoldt *et al*., 1997). Other members of the family are involved in repression of pseudohyphal growth in *S. cerevisiae* (Ward *et al*., 1995), conidiation in *Aspergillus nidulans* and *Penicillium marneffii* (Aramayo *et al*., 1996; Borneman *et al*., 2002) and the formation of aerial hyphae and conidiation in *Glomerella cingulata* (Tong *et al*., 2007) and *Wangiella dermatitidis* (Wang and Szaniszlo, 2007).

We describe here the role of Efg1 in *Candida parapsilosis*, a major human fungal pathogen that is a member of the CTG clade and is particularly associated with infection of premature neonates (Trofa *et al*., 2008; van Asbeck *et al*., 2009). *C. parapsilosis* grows in yeast and pseudohyphal forms but not as true hyphae, and although variations in colony morphology have been observed (Lott *et al*., 1993; Enger *et al*., 2001; Laffey and Butler, 2005; Kim *et al*., 2006), no equivalent of white-opaque switching has been identified. The *C. parapsilosis* species group also appears to be completely asexual, and do not exhibit the parasexual cycle observed in *C. albicans*, *C. dubliniensis* and *C. tropicalis* (Bennett and Johnson, 2005; Butler, 2010; Porman *et al*., 2011; Sai *et al*., 2011). We show that Efg1 represses a morphological switch from concentric to smooth colony formation, and is also a repressor of filamentation in hypoxic conditions. We use a combination of RNA-seq and ChIP-seq to identify Efg1 targets. Efg1 directly regulates a significant number of transcription factors, particularly those with long promoter sequences. Targets of Efg1 are also enriched in binding sites of other transcription factors. In addition, we show that Efg1 is required for biofilm development, and for virulence.

## Results

### *Deleting EFG1 in* C. parapsilosis

The *EFG1* orthologue in *C. parapsilosis* was identified using sequence comparison and synteny conservation (Fitzpatrick *et al*., 2010). Both alleles in the diploid were deleted using three different approaches. First, the alleles were replaced with a recyclable nourseothricin-resistant marker as described in Ding and Butler (2007). Second, each allele was replaced with either *LEU2* (from *Candida maltosa*) or *HIS1* (from *C. dubliniensis*) in an auxotrophic background strain. The auxotrophic markers were amplified using either a long oligonucleotide (approach 2) or by fusion PCR (approach 3), using methods similar to those described by Noble and Johnson (2005) (Fig. S1). *EFG1* was re-introduced into one deletion strain under the control of the *ACT1* promoter as shown in Fig. S1D. All phenotypes were tested using knockouts from each approach to ensure that they result from the loss of the *EFG1* gene and not to a secondary effect.

**Figure 1:**
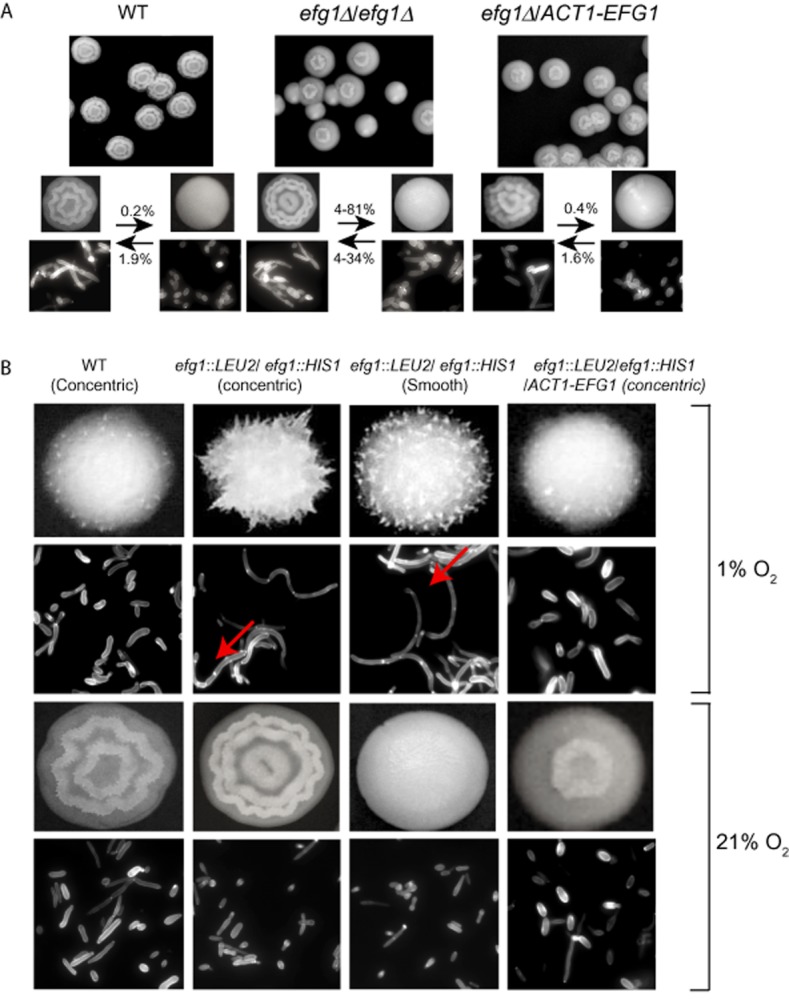
Efg1 regulates colony switching in *C. parapsilosis*. Both alleles of the *EFG1* gene were deleted as described in Fig. S1.A. Wild-type (CLIB214) cell cultures consist of mostly concentric colonies, whereas *efg1*Δ*/* *efg1*Δ (LCP7 is shown here) have a mixture of concentric and smooth colonies. The rate of switching between concentric and smooth cells varies, but can be as high as 81%, with a substantial number of sectored colonies (Table S1). Re-introducing *EFG1* reduces the switching rate (LCP7_RI). Cells from concentric colonies tend to be elongated, whereas those from smooth colonies are more round and yeast-like.B. Wild-type colonies are generally smooth in hypoxic conditions (1% oxygen), and do not form the concentric phenotype obvious in normoxia (21% oxygen). Deleting both alleles of *EFG1* (LCP7 is shown here) results in rough and spiked colonies in hypoxic conditions, with an increase in elongated and pseudohyphal cells (Table S2). The arrows indicate the cell wall between a mother cell and a pseudohyphal bud. The reconstituted strain is more similar to the wild-type phenotype. In normoxic conditions, few pseudohyphae are observed. The top panels show colonies and the bottom panels show corresponding calcofluor white-stained cells for growth at 1% and 21% oxygen.

The most obvious result of deleting *EFG1* is that it leads to a dramatic increase in the level of morphological switching observed at the colony level (Fig. 1A). Almost all colonies of the *C. parapsilosis* type strain (CLIB214) are a ‘wrinkled’ or ‘concentric’ morphology (composed of a series of concentric rings). Switching to other morphologies occurs at a very low level (Lott *et al*., 1993; Enger *et al*., 2001; Kim *et al*., 2006). However, the *efg1* deletion consists of a mixture of both concentric and smooth colonies (Fig. 1A). When grown in culture, concentric colonies give rise to > 10% of smooth colonies and smooth colonies to > 12% concentric colonies. The switching rate varies in the different knockouts (Table S1) but is always significantly higher than the wild type, which has a switching rate of approximately 0.2% (concentric to smooth) and 2% (smooth to concentric). Two of the *efg1* knockouts (generated using the fusion PCR approach) switch from concentric to smooth colonies at a very high rate (65–81%). None of strains deleted for just one *EFG1* allele exhibited an increased switching rate (not shown). We reintroduced *EFG1* expressed from the *ACT1* promoter into one of the strains with the highest switching rate (Fig. S1). *EFG1* expression from this construct is approximately the same level as in a heterozygous *EFG1/efg1* deletion strain (data not shown), and switching is returned to wild-type levels (Fig. 1A, Table S1).

Individual cells of *C. parapsilosis* (particularly those derived from the type strain CLIB214) are more elongated than *C. albicans* or *S. cerevisiae* yeast cells (Fig. 1A and B). However, we found that cells from the smooth colonies tend to be more round and yeast like when grown in normal atmospheric conditions (Fig. 1, Table S2). Because *EFG1* is reported to be a repressor of filamentation in hypoxia in *C. albicans*, we also examined the morphology of the *C. parapsilosis efg1* knockouts in low-oxygen conditions. As shown in Fig. 1B, growth in hypoxia induces a novel ‘spiked’ morphology in colonies that is not observed in the wild type, irrespective of the concentric/smooth morphology, independent of the level of oxygen available. However, when *EFG1* is deleted, there is a significant increase in the number of pseudohyphal cells in hypoxic conditions (Fig. 1B, Table S2). Long chains of cells are very evident. The structures are reminiscent of hyphae, except that there is a cell wall between the mother (yeast) cell and the first pseudohyphal bud (indicated with arrows in Fig. 1B). Cells grown in liquid culture have the same phenotype (not shown). The phenotype of colonies of the *efg1/ACT1-EFG1* complemented strain is more similar to the wild type, and the percentage of pseudohyphal cells is between that of the wild-type and the deletion strain (Table S2).

### Deleting EFG1 affects biofilm development

*Candida parapsilosis* grows as biofilms on indwelling medical devices (Hawser and Douglas, 1994; Silva *et al*., 2009; Melo *et al*., 2011). The biofilm architecture is substantially different from that observed for *C. albicans*, although some of the same regulators are required. For example, the transcription factor Bcr1 is required for biofilm development in both species (Nobile *et al*., 2006; Ding and Butler, 2007; Finkel and Mitchell, 2011). However, the mechanism and targets are likely to be different; the CFEM family members are targets of Bcr1 in both, but have a role in biofilm development only in *C. albicans* (Ding and Butler, 2007; Ding *et al*., 2011).

**Figure 2:**
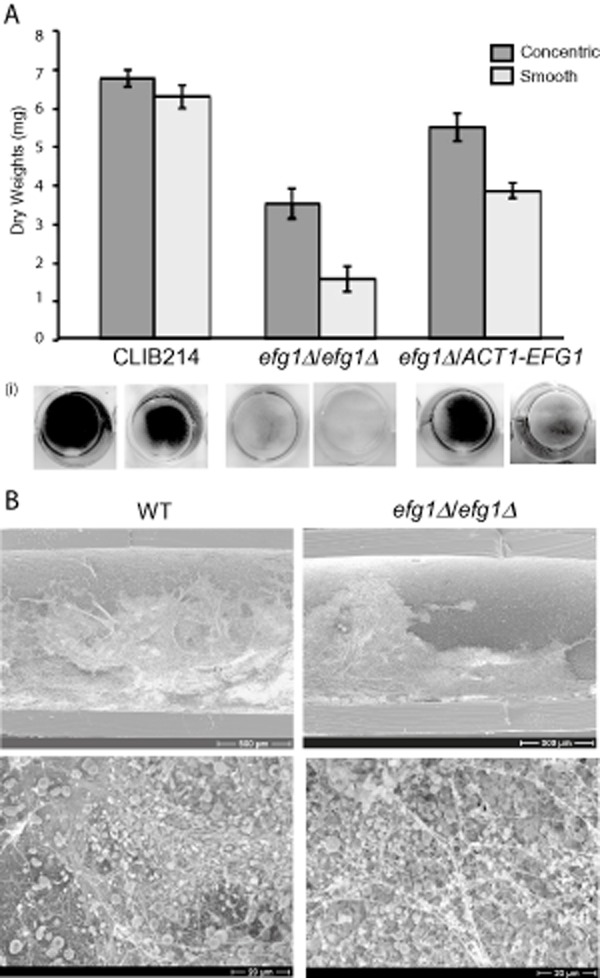
Efg1 regulates biofilm development by *C. parapsilosis*.A. The *in vitro* biofilm mass of smooth and concentric colonies from wild type (CLIB214), *efg1*Δ*/efg1*Δ deletion strains (including a mixture of strains LCP2, LCP5 and LCP8) and the reconstituted strain LCP7_RI was determined by measurement of dry weights. The average ± standard deviation from three independent measurements is shown. The panels below show biofilm growth on 24-well Nunc plates stained with crystal violet.B. *In vivo* biofilms were developed in the rat catheter model. Concentric wild-type (CLIB214) cells and smooth *efg1/efg1* deletion (LCP1) cells were allowed to develop for 24 h and then visualized by SEM at two magnifications.

Efg1 is required for biofilm formation by *C. albicans* (Ramage *et al*., 2002; Nobile *et al*., 2012). We therefore tested its role in *C. parapsilosis*. Deleting *EFG1* in *C. parapsilosis* reduces biofilm development irrespective of colony morphology (Fig. 2). Both smooth and concentric colonies carrying an *efg1* deletion have reduced biofilm formation on the surface of 24-well Nunc plates compared with wild type, with smooth cells having a slightly more pronounced defect. The phenotype is partially restored by re-introducing *EFG1*. The differences were observed using dry weight measurements of mature biofilms, and also crystal violet staining of adhered cells (Fig. 2A). We also determined the effect of deleting *EFG1* on biofilms developed *in vivo*, using one deletion strain (LCP1) in the rat catheter model developed by Andes *et al*. (2004). As shown in Fig. 2B, *in vivo* biofilms generated by smooth cells carrying an *efg1* deletion are mildly reduced. Concentric colonies deleted for *EFG1* were not tested *in vivo*.

### Efg1 affects cell wall function

**Figure 3:**
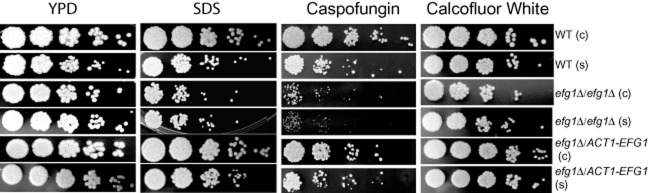
Deleting Efg1 increases sensitivity to cell wall stress. Three microlitres from serial dilutions in PBS was inoculated on YPD alone, and YPD with SDS (0.04%), caspofungin (0.1 μg ml^−1^) and calcofluor white (12 μM). Strains include concentric (c) and smooth (s) colonies from wild type (*C. parapsilosis* CLIB214), *efg1*Δ*/efg1*Δ deletion (LCP8) and the reconstituted strain (LCP7_RI).

In *C. albicans*, Efg1 is a major regulator of cell wall genes (Sohn *et al*., 2003; Zavrel *et al*., 2012). We therefore tested the phenotype of the *efg1* knockouts under conditions of known cell wall stress. Figure 3 shows that knocking out *EFG1* confers sensitivity to SDS and caspofungin, and reduces growth on calcofluor white. Both smooth and concentric cells are more susceptible to stress, but the phenotype of smooth cells is more dramatic.

### Efg1 is required for virulence in C. parapsilsosis

In *C. albicans*, deleting *EFG1*, either alone or in combination with the transcription factor *CPH1*, results in greatly attenuated virulence in infection of mice (Lo *et al*., 1997), *Caenorhabditis elegans* (Pukkila-Worley *et al*., 2009), *Drosophila* (Chamilos *et al*., 2006) and zebrafish (Chao *et al*., 2010). We determined the effect of deleting *EFG1* on the virulence of *C. parapsilosis*, using the insect *Galleria mellonella*, which has been shown to be a good model for candidiasis (Fallon *et al*., 2012).

**Figure 4:**
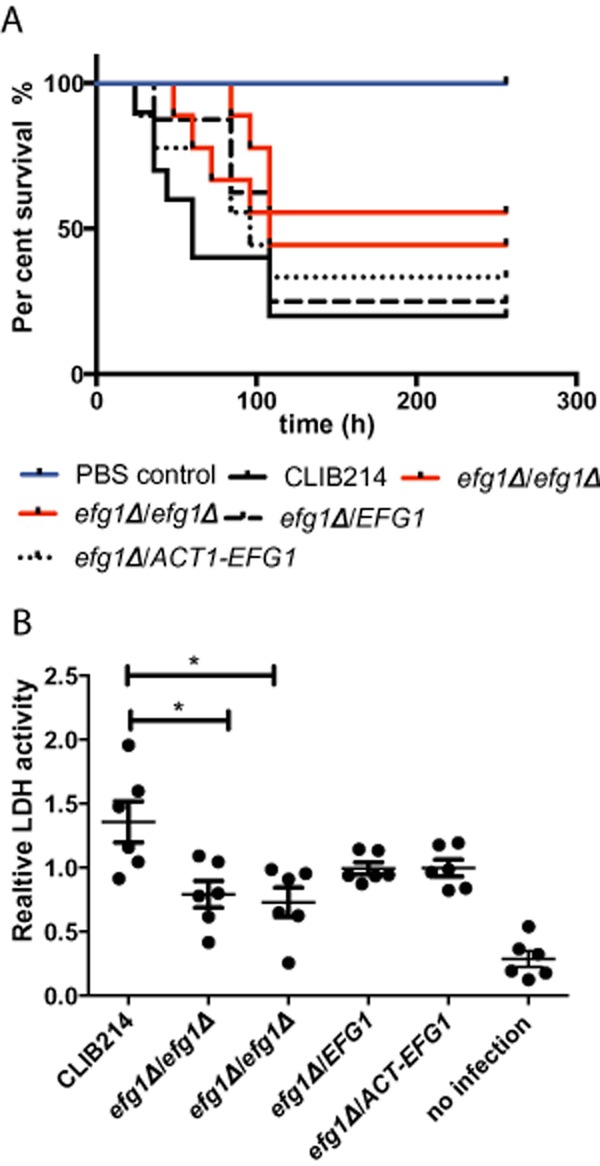
Efg1 is required for virulence.A. *G. mellonella* larvae were inoculated with 2 × 10^6^ yeast cells in PBS, and survival was monitored over time. The strains tested included wild type (CLIB214), *efg1*Δ*/efg1*Δ deletion strains (LCP5 and LCP7), a heterozygote *efg1*Δ/*EFG1* (LCPH1) and the reintegrant *efg1/ACT1-EFG1* strain LCP7_RI. The survival curves for the *efg1*Δ*/efg1*Δ deletion strains are significantly different from the curves for the wild-type infections (Mantel-Cox test, P-value < 0.01).B. Human PBMC-derived macrophages from four independent donors were co-cultured with *C. parapsilosis* cells, and the relative levels of LDH in the media were determined after 48 h. The strains used are the same as in (A). The levels of damage induced by the *efg1*Δ*/efg1*Δ deletion strains is significantly less than that of the wild type (two-tailed Wilcoxon text, *P*-value < 0.05).

We first confirmed that *C. parapsilosis* wild-type strains are capable of killing *G. mellonella* larvae. Following infection, survival is reduced to approximately 20% within 100 h, compared with 100% survival for uninfected larvae (Fig. 4A). Deleting *EFG1* however significantly attenuates virulence (Fig. 4A – two independent deletions were tested). Interestingly, strains carrying only one intact *EFG1* allele (either deleted for only one allele, or carrying only the *ACT1-EFG1* allele) have virulence levels between the wild-type and the double-deletion strains (Fig. 4A). This is similar to the intermediate biofilm phenotype described in Fig. 1, and suggests that similar to *C. albicans*, deleting one allele of *EFG1* results in a haploinsufficiency phenotype (Zavrel *et al*., 2012).

To determine if deleting *EFG1* also affects virulence in mammalian systems, we tested the ability of *C. parapsilosis* to inflict damage on human PBMC-DM (peripheral blood mononuclear cell-derived macrophages) (Fig. 4B). Damage was measured by the release of Lactate Dehydrogenase (LDH). Deleting *EFG1* reduces the release of LDH after 24 h (not shown) and 48 h (Fig. 4B). Once again, having only one intact *EFG1* allele results in a phenotype that is intermediate between the wild type and *efg1/efg1* deletions.

### Identifying the targets of Efg1

We used a combination of chromatin immunoprecipitation with next-generation sequencing (ChIP-seq) to identify promoters that are directly bound by Efg1. We generated a tagged version of Efg1 in a strain carrying a deletion of one *EFG1* allele. A MYC epitope tag was introduced just 5′ to the stop codon of Efg1 by homologous recombination, using tools developed for *C. albicans* (Lavoie *et al*., 2008). Two independent constructs were confirmed by PCR (Fig. S1). The strains carrying the tagged allele remain in the concentric morphology, indicating that the construct is biologically functional.

The immunoprecipitated bound targets were investigated by next-generation sequencing using Illumina technology. Reads were mapped to the current annotation of the *C. parapsilosis* genome (Guida *et al*., 2011) using BWA (Li and Durbin, 2009). Three experimental samples (from two independently tagged strains) and three control (input) samples were used. Enriched peaks were identified using MACS2 (Zhang *et al*., 2008; Feng *et al*., 2012). We used estimates of the Irreproducible Discovery Rate (IDR) to incorporate statistical measurements of reproducibility from multiple replicate samples (Landt *et al*., 2012). A list of bound regions [ranked by π score, fold change and log_10_ of the adjusted *P* value (Xiao *et al*., 2012)] is shown in Table S3.

**Figure 5:**
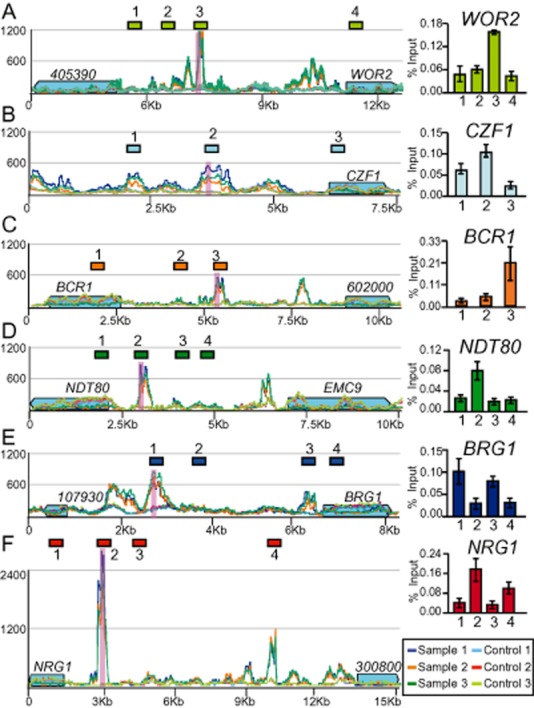
Binding of Efg1 to intergenic regions. (A)–(F): each represent a different intergenic region, with the coding sequences marked by blue boxes where the arrowhead indicates the direction of transcription. Short gene names are given where possible; long numbers (e.g. 405390) should be preceded by the identifier CPAR2. (A) *WOR2/CPAR2_405390*; (B) *CZF1*; (C) *BCR1/CPAR_60200*; (D) *NDT80/ECM9*; (E) *BRG1/CPAR2_107930*; (F) *NRG1*/*CPAR2_300800*. The scale shows the coverage range. Different coloured lines indicate the mapped reads from three sample (Efg1-myc) and three control (input) lanes. The peak with the highest summit fold change relative to the input in each intergenic region is indicated in pink. The images were constructed using the Integrative Genomics Viewer (IGV) (Robinson *et al*., 2011). Sequences within each intergenic region (indicated by numbered coloured boxes) were chosen for analysis by ChIP-PCR. The enrichment of PCR products in immunoprecipitated samples as a % of the input samples is plotted. The average ± standard deviation from three independent measurements is shown.

Efg1 binds over large distances in intergenic regions, often with more than one peak per region (Fig. 5). For example, Efg1 extends over at least > 5 kb in the promoter sequence of *CZF1*. For ranking purposes we chose a single peak within each intergenic sequence, with the highest ranking (π score, highlighted in pink in Fig. 5). The experiments were highly reproducible, as shown by the different colour tracks in Fig. 5. We also confirmed the binding sites for six promoters using ChIP-PCR (Fig. 5).

Of 1246 peaks identified, 53 lie within coding sequences and 38 are 3′ to two open reading frames, and were not included in subsequent analyses. The remaining 1155 peaks were assigned to 931 promoters. Because 395 of these regions are within divergent promoters, up to 1326 genes may be directly controlled by the transcription factor. Gene Ontology analysis indicates that the target genes are significantly enriched for regulatory proteins, including transcription factors (*P* value = 3.47e-7, Table S3C). At least 93 transcription factors are direct targets, including several previously shown to be part of networks with Efg1 that regulate white-opaque switching and biofilm development in *C. albicans* (Zordan *et al*., 2007; Nobile *et al*., 2012), and others associated with hyphal growth (Lassak *et al*., 2011) (Fig. 5).

**Figure 6:**
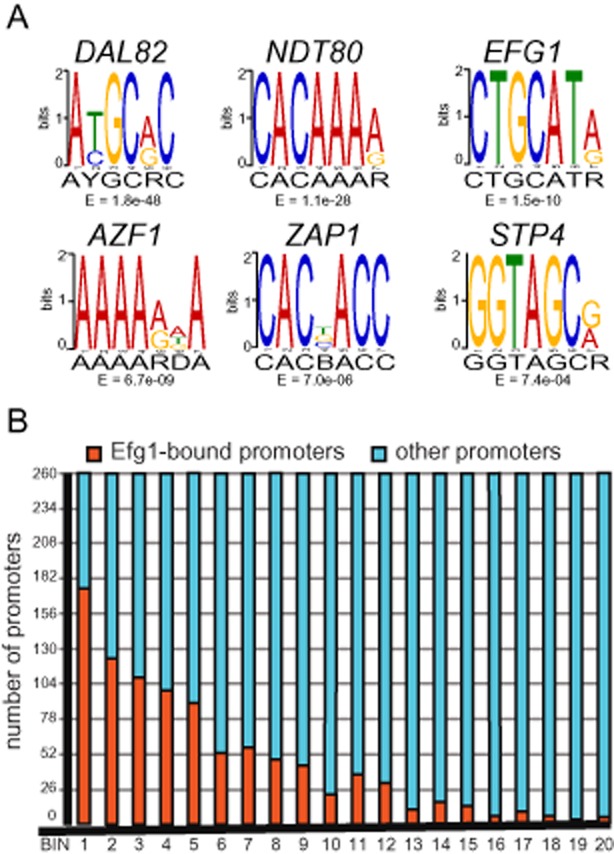
Identification of conserved motifs in Efg1-bound regions.A. Enriched motifs in Efg1-bound intergenic sequences were identified using DREME (Bailey, 2011). All significant motifs are shown, with the most likely associated regulatory protein.B. A total of 5200 intergenic regions from *C. parapsilosis* were divided into 20 bins depending on decreasing promoter length. Each bin contains 260 intergenic regions. Bin1 contains intergenic regions ranging from 2.7 kb to 11.5 kb, and bin 20 contains promoters ranging from 81 bp to 109 bp. The subset of intergenic regions bound by Efg1 are highlighted in orange.

We used DREME (Bailey, 2011) and FIMO (Grant *et al*., 2011) to identify conserved motifs and therefore the most likely binding site of *EFG1* in *C. parapsilosis*, using 100 bp regions around the peak summits from the ChIP-seq experiments. Six significant motifs were identified from both analyses of all 931 promoters (Fig. 6). The most significant motifs (AYGCRC and CACAAAR) most closely resemble the binding sites for Dal82 and Ndt80 in *S. cerevisiae* (Dorrington and Cooper, 1993; Zhu *et al*., 2009) (Fig. 6A). The third most significant motif (CTGCATR) resembles the binding site for Sok2 and Phd1 from *S. cerevisiae* (TGCAGNNA) (Harbison *et al*., 2004), which are co-orthologues of Efg1. The motif is also similar to the predicted Efg1 binding sites from *C. albicans* recently reported by Lassak *et al*. (2011) (TATGCATA) and Nobile *et al*. (2012) (RTGCATRW). The remaining motifs are similar to binding sites for transcription factors with zinc-finger DNA-binding domains, including Zap1, which has been implicated in regulation of biofilm development in *C. albicans* (Nobile *et al*., 2009). All six motifs are present in 40% of the intergenic regions, putative Efg1 and Ndt80 binding sites are found together in 70%, and Efg1, Ndt80 and Dal82 sites co-occur in 65% (Table S4). It is therefore likely that the targets of Efg1 in *C. parapsilosis* are also regulated by other transcription factors.

The intergenic regions bound by Efg1 are significantly longer than the average intergenic region in *C. parapsilosis* (average of 1.7 kb compared with 0.6 kb, *P* value < 2.2 e-16, Welch's *t*-test, Fig. 6B). Nobile *et al*. (2012) previously found that intergenic regions bound by biofilm regulators in *C. albicans* are larger than average, and suggested that the combination of short regulatory motifs with larger targets for mutations may allow new genes to be incorporated in a regulatory network.

### Identification of the EFG1 regulon

To evaluate the overall biological role of Efg1, we determined the transcriptional profile of *efg1* knockout concentric and smooth cells and compared them to the profile of wild-type (concentric) cells. We profiled two independent deletion strains, and included two smooth and three concentric samples (Table S5). We compared the expression of concentric wild-type cells to *efg1* deletions in both concentric and smooth morphologies (Table S5A). However, to ensure that the differences we observed are related to the gene deletion rather than to colony type, we predominantly analysed the comparison of concentric cells from both wild-type and *efg1* deletion strains.

A total of 353 genes are differentially regulated in concentric cells from the *efg1* knockout (Table S5). Efg1 acts as both an activator and a repressor of gene expression; expression of 167 genes is reduced in the *efg1* deletions, and expression of 186 genes is increased. Approximately 53% of the genes that are differentially expressed (and 60% of the downregulated genes) are also bound by Efg1 (Table S6A). Similar to *C. albicans* (Tebarth *et al*., 2003), Efg1 negatively regulates its own expression. When the open reading frame is deleted, expression of the 5′ UTR region is increased in both concentric and smooth cells (Fig. S2). Upregulated genes are enriched in processes associated with ergosterol metabolism (Table S5). However, the promoter regions of most of these genes are not bound by Efg1, and so they are likely to be regulated indirectly.

There are significant differences in expression patterns between the smooth and concentric colonies from *efg1* deletions; 380 genes are differentially expressed (Table S5B). Genes upregulated in concentric cells are enriched in processes associated with iron metabolism, whereas downregulated genes are enriched in metabolism of amino acid and organic acids. Less than 30% are bound by Efg1, suggesting that differential expression is likely to be related to morphology, rather than to deletion of *EFG1*.

Expression of some of the genes with the largest decreases in expression (which are also bound by Efg1) were confirmed using quantitative PCR. Expression is reduced in both concentric and smooth cells (Fig. S3).

### Comparison of Efg1 targets in C. parapsilosis and C. albicans

**Figure 7:**
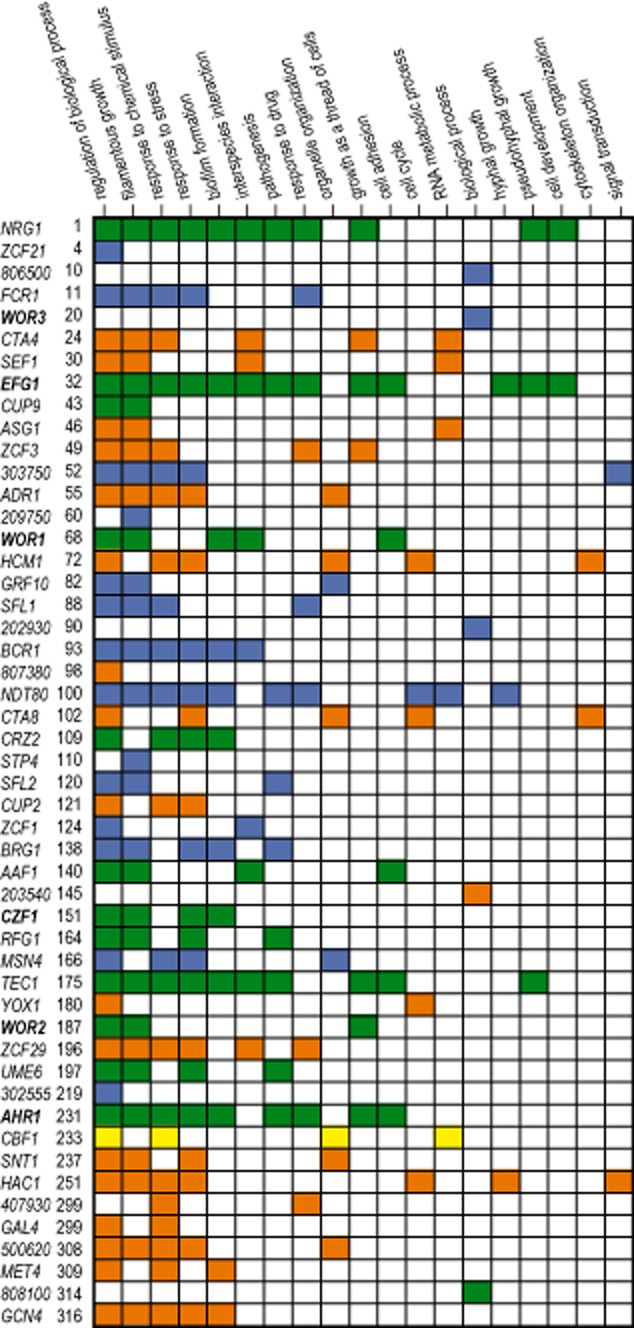
Similarities and differences between targets of Efg1 in *C. parapsilosis* and *C. albicans*. The top 50 transcription factors bound by Efg1 in *C. parapsilosis* are listed. The names of the *C. albicans* orthologues were used where possible; six-digit gene names (missing the suffix ‘CPAR2’) are used where only a *C. albicans* generic identifier is available (i.e. orf19). The gene name is followed by the rank from the ChIP-seq analysis (Table S2A). Processes associated with the 50 genes were determined using GO Slim Mapper at the Candida Genome Database (Inglis *et al*., 2012). Efg1 targets conserved in *C. albicans* are shown in green [identified by both Lassak *et al*. (2011) and Nobile *et al*. (2012)], yellow [identified by Lassak *et al*. (2011) only] and blue [identified by Nobile *et al*. (2012) only]. Orange boxes indicate that the relevant transcription factor is bound by Efg1 in *C. parapsilosis* only. Only processes including at least two genes were included. Genes highlighted in bold are part of the circuit regulating the white/opaque switch in *C. albicans*.

We compared our ChIP-seq analysis from *C. parapsilosis* to the ChIP-chip analysis used to identify Efg1-bound genes in *C. albicans* reported by Nobile *et al*. (2012) and Lassak *et al*. (2011). A total of 218 bound genes are shared with the *C. albicans* data set from Nobile *et al*. (2012) and 29 with Lassak *et al*. (2011) (Table S7, Fig. 7). The shared targets are enriched for processes associated with filamentous growth, biofilm development, and cell adhesion (Table S7C, Fig. 7). Many of the shared targets are transcription factors (Fig. 7), suggesting that some regulatory networks are conserved in the two species, even if the biological processes (such as filamentation) are not.

Nobile *et al*. (2012) suggested that the biofilm regulatory network has arisen recently in *C. albicans*. We confirmed that the targets of Efg1 in *C. albicans* are enriched for young genes, using a very restricted definition of genes that appeared on the *C. albicans/C. dubliniensis/C. tropicalis* branch (*P* value = 1.0e-4; Table S3C). In contrast, genes bound by Efg1 in *C. parapsilosis* are not significantly enriched for young genes (i.e. those arising in the *C. parapsilosis/C. orthopsilosis/Lodderomyces elongisporus* lineage). The role of Efg1 in *C. parapsilosis* may therefore resemble the ancestral role, rather than that observed in *C. albicans*.

We attempted to compare the genes differentially expressed in an *efg1* deletion in *C. parapsilosis* with those differentially expressed in an equivalent knockout in *C. albicans* (Nobile *et al*., 2012). However, we identified only 353 differentially expressed genes from *C. parapsilosis* concentric cells, whereas Nobile *et al*. (2012) identified > 3000 in *C. albicans*, corresponding to half the genome. Part of the difference is probably because the profiles described by Nobile *et al*. (2012) are from biofilm cells. In contrast, Doedt *et al*. (2004) detected only 283 genes differentially expressed in a *C. albicans efg1* deletion grown in similar conditions to ours. Approximately 80 are also differentially expressed in *C. parapsilosis* but some have different patterns of expression (e.g. downregulated in *C. albicans efg1* and upregulated in *C. parapsilosis efg1*) (Table S5D).

The comparison of both Efg1-bound and differentially expressed genes in *C. albicans* and *C. parapsilosis* suggests that some pathways are conserved between the two species, and some are not. Many of the shared components are transcription factors, including orthologues associated with biofilm development (e.g. *BCR1*, *NDT80*, *BRG1*, *TEC1*), white-opaque switching (e.g. *WOR1, WOR2*, *CZF1*) and filamentation (e.g. *NRG1, SFL1, UME6*) in *C. albicans* (Liu, 2001; Bauer and Wendland, 2007; Zordan *et al*., 2007; Nobile *et al*., 2012; Lohse *et al*., 2013) (Fig. 7). However, there are also substantial differences, particularly in the RNA-seq profiles, suggesting that many of the targets are species-specific.

### Efh1 shares few functions with Efg1

*Candida albicans* contains a paralogue of *EFG1*, called *EFH1*, that is also a member of the APSES family (Doedt *et al*., 2004). We identified an orthologue of *EFH1* in *C. parapsilosis* (CPAR2_104660), and deleted both alleles in two independent strains using the fusion PCR method (Fig. S1). Deleting *EFH1* does not affect biofilm development, or growth in the presence of cell wall stress (Fig. S4). The deletion does not affect switching from concentric to smooth cells, although a novel colony phenotype does arise at a low level (approximately 3%, Fig. S4, Table S1).We also found that deleting both *efg1* and *efh1* resulted in a phenotype that is indistinguishable from *efg1* deletion alone for biofilm development and sensitivity to cell wall drugs (Fig. S4), and for cell morphology during growth in normoxic and hypoxic conditions (Table S2).

## Discussion

Efg1 is a major regulator of morphogenesis in fungal species, originally characterized in *C. albicans* as a regulator of filamentous growth (Stoldt *et al*., 1997). *S. cerevisiae* has two Efg1 co-orthologues, called Phd1 and Sok2, and both are involved in the regulation of pseudohyphal growth (Gimeno and Fink, 1994; Ward *et al*., 1995; Pan and Heitman, 2000). The proteins are members of the APSES family, which contain a novel conserved DNA-binding domain (KilA-N domain) related to a basic helix-loop-helix domain that is conserved in many eukaryotic and bacterial viruses (Marchler-Bauer *et al*., 2011). Other members such as StuA in *A. nidulans* and Asm-1 in *Neurospora crassa* (filamentous ascomycetes, or Pezizomycotina) also control yeast-hyphal transitions (Miller *et al*., 1992; Aramayo *et al*., 1996). An orthologue from a basidiomycete, *Ustilago maydis*, has recently been associated with regulation of dimorphic growth in this species (Garcia-Pedrajas *et al*., 2010). However, in other species, the APSES proteins do not control dimorphism, but instead regulate processes such as mating and conidiation (Borneman *et al*., 2002). A role for APSES proteins in regulation of development has therefore been conserved across fungal species, but Borneman *et al*. (2002) suggest that regulation of hyphal growth is restricted to species that divide by budding. This is supported by our analysis; deleting *EFG1* in *C. parapsilosis* increases pseudohyphal growth in hypoxic conditions (Fig. 1B, Table S2).

**Figure 8:**
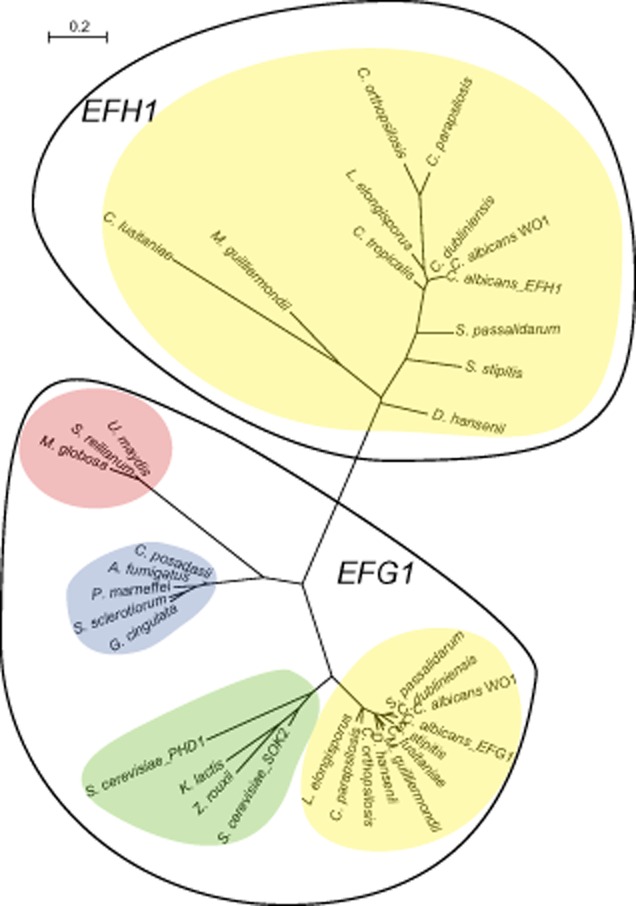
Conservation of Efg1-like proteins in fungi. Sequences were aligned using SeaView (Gouy *et al*., 2010), and an unrooted phylogenetic tree was constructed from the conserved bHLH domains by implementing the PhyML algorithm within SeaView. The CTG clade species are highlighted in yellow, *Saccharomyces* species in green, Pezizomycotina (filamentous ascomycetes) species in purple, and Basidiomycetes in peach. Gene names/accession numbers: *Ustilago maydis* um15042, *Sporisorium reilianum* CBQ71974, *Malassezia globosa* MGL_0313, *Coccidioides posadasii* XP_003066203, *Aspergillus fumigatus* XP_755125, *Penicillium marneffei* XP_002146488, *Sclerotinia sclerotiourum* XP_001590416, *Glomerella cingulata* ABQ43363, *Saccharomyces cerevisiae SOK2* and *PHD1*, *C. albicans* SC5314 orf19.8243 and orf19.5498, *C. albicans* WO1 CAWG_02083 and CAWG_04378, *C. dubliniensis* CD36_33560 and CD36_20730, *C. tropicalis* CTRG_01780, *C. parapsilosis* CPAR2_701620 and CPAR2_104660, *C. orthopsilosis* CORT0G01760 and CORT0B05860, *L. elongisporus* LELG_05390 and LELG_01890, *Spathaspora passalidarum* SPAPADRAFT_59209 and SPAPADRAFT_66101, *Scheffersomyces stipitis* PICST_67427 and PICST_32309, *Meyerozyma guilliermondii* PGUG_03651 and PGUG_02282, *Clavispora lusitaniae* CLUG_02047 and CLUG_01623.

Both Efg1 and Efh1 in *C. albicans* have KilA-N/APSES domains (Doedt *et al*., 2004). However, unlike the two proteins in *S. cerevisiae* (Phd1 and Sok2), Efg1 and Efh1 fall into different clades (Fig. 8). *PHD1* and *SOK2* arose from an ancient duplication event in the Whole Genome Duplication clade containing *S. cerevisiae* (Wolfe and Shields, 1997), and both are orthologous to *C. albicans EFG1* (Fig. 8). The Efg1/Efh1 proteins are poorly conserved throughout their entire lengths, and contain several polyQ regions that may be associated with transcriptional activation. However, the DNA-binding domains (KilA-N domain) are well conserved, and were used to construct the phylogenetic tree shown in Fig. 8. Whereas both Efg1 and Efh1 are present in all the *Candida* species, the Efg1 paralogues are similar to the APSES proteins from other fungal species, and the Efh1 proteins cluster in a separate clade that appears to be unique to *Candida* (Fig. 8). The Efh1 proteins are also evolving rapidly, as shown by the long branch lengths in the Efh1 cluster in Fig. 8, whereas the DNA-binding domains of Efg1 are remarkably similar (more similar than proteins within the *Saccharomyces* clade for example).

The rapid evolution of the Efh1 proteins makes it very difficult to determine their origin [due to difficulties with long-branch attraction (Fares *et al*., 2006)]. However, it is more likely that Efh1 arose in a common ancestor of the *Candida* species, rather than an earlier origin with subsequent loss in other fungal clades. The function of Efh1 remains to be determined. *EFH1* has little effect on the Efg1-dependent phenotypes in *C. albicans*, except that a double *efg1/efh1* deletion forms lateral hyphae faster and is more filamentous in hypoxic conditions, and that pseudohyphal formation caused by overproduction of *EFH1* requires *EFG1* (unlike filamentation caused by overproduction of *EFG1*, which is independent of *EFH1*) (Doedt *et al*., 2004). Efg1 and Efh1 share very few regulatory targets in *C. albicans* (Doedt *et al*., 2004), and we have shown that in *C. parapsilosis*, *EFH1* does not regulate response to stress or biofilm development, and the phenotype of a double *efg1/efh1* mutant is indistinguishable from an *efg1* deletion. The switch from concentric to smooth colony morphology is also not increased by deleting *EFH1*, although a novel colony phenotype is observed at a very low rate (Fig. S4). *EFH1* regulates colonization of the murine intestinal tract by *C. albicans* in a commensal model of infection (White *et al*., 2007); any similar role in *C. parapsilosis* has not been tested.

High-frequency phenotypic switching was first observed in *Candida* species in the 1930s (Negroni, 1935; Soll, 2002). The best-characterized system is the white-opaque switch in *C. albicans*; switching to opaque cells is required for efficient mating (Slutsky *et al*., 1987; Miller and Johnson, 2002). White and opaque cells have different transcriptional and metabolic profiles, they respond differently to phagocytosis, and they exhibit different colonization and virulence properties (reviewed in Morschhauser, 2010). Switching is a stochastic process, determined by the levels of the master regulator Wor1 (Huang *et al*., 2006; Srikantha *et al*., 2006; Zordan *et al*., 2007). Efg1 is a major component of the regulatory network (Srikantha *et al*., 2000; Doedt *et al*., 2004; Zordan *et al*., 2007). Switching is also affected by chromatin modification (Klar *et al*., 2001; Srikantha *et al*., 2001; Hnisz *et al*., 2009). White-opaque switching is probably restricted to *C. albicans, C. dubliniensis* and *C. tropicalis* (Pujol *et al*., 2004; Porman *et al*., 2011).

Other high-frequency switching systems have been reported in *Candida* species; for example in *C. albicans* 3135A, eight different interchangeable colony morphologies were described (Slutsky *et al*., 1985; Ramsey *et al*., 1994). Expression of some known virulence factors such as phospholipases and proteinases varies in the different phenotypes (Antony *et al*., 2009). Switching between unusual colony morphologies has also been reported in *C. tropicalis*, *C. parapsilosis* and *C. lusitaniae* (Enger *et al*., 2001; Miller *et al*., 2006; Franca *et al*., 2011), and outside the CTG clade, in *Candida glabrata* and *Candida krusei* (Lachke *et al*., 2000; Arzmi *et al*., 2012). However, the underlying regulatory mechanisms have not been elucidated. We showed here that Efg1, a major regulator of white-opaque switching in *C. albicans*, also regulates colony morphology in *C. parapsilosis*. The rate of switching varies between the various *efg1* deletion strains (Table S1), but even the strains with high switching rates return to wild-type levels when *EFG1* is re-introduced. Concentric and smooth cells deleted for *EFG1* have substantially different expression profiles, enriched for genes associated with iron metabolism (Table S5). It is therefore likely that two colony morphologies differ in metabolism, similar to white/opaque cells of *C. albicans*.

Efg1 is required for biofilm development in *C. albicans*, both in *in vitro* and in *in vivo* models (Ramage *et al*., 2002; Ricicova *et al*., 2010; Nobile *et al*., 2012). Unlike many other biofilm regulators, Efg1 is required for biofilm development in low oxygen and high carbon dioxide, conditions that mimic systemic infection (Stichternoth and Ernst, 2009). We have shown that deleting *EFG1* in *C. parapsilosis* also reduces biofilm formation *in vitro* (Fig. 2A). In hypoxic conditions wild-type strains make very little biofilm, and this is further reduced in the *efg1* deletions (not shown). The defect *in vivo* is not as pronounced; the view of a catheter at low magnification shows that the overall mass is somewhat reduced, but the structure of the biofilm is essentially unchanged (Fig. 2B). One of the major differences between *C. parapsilosis* and *C. albicans* is that the former does not form true hyphae. Biofilms formed by *C. albicans* consist of a mixture of yeast, pseudohyphal and hyphal cells; the yeast cells form a basal layer, with hyphae in the upper layers of the mature biofilm (Baillie and Douglas, 1999; Blankenship and Mitchell, 2006; Ramage *et al*., 2009). *C. parapsilosis* biofilms in contrast consist of yeast and pseudohyphal cells only (Ding and Butler, 2007; Ramage *et al*., 2009). *C. albicans* strains with an *EFG1* deletion are locked in yeast phase in most growth conditions, including in biofilms (Ramage *et al*., 2002), and other mutants with reduced hyphal development also have reduced biofilm mass (Baillie and Douglas, 1999). Although the entire biofilm network identified by Nobile *et al*. (2012) does not appear to be dependent on hyphal growth, it is very likely that for the *EFG1* deletion some of the reduction in biofilm development is due to reduced hyphal formation. The lack of hyphae in *C. parapsilosis* may partly explain the difference in phenotype between the *EFG1* deletions in the two species *in vivo*, where hyphal growth is likely to be more important for *C. albicans* biofilms.

We found that deleting *EFG1* in *C. parapsilosis* results in attenuated virulence in an insect model, and reduces damage of human macrophages (Fig. 4). In *C. albicans*, reduction in virulence of an *efg1* deletion has generally been related to a failure of the knockout to grow as hyphae (Lo *et al*., 1997; Chamilos *et al*., 2006; Pukkila-Worley *et al*., 2009). However, even in models where *efg1* strains do form hyphae (such as gnotobiotic mice deficient in natural killer and T cells) virulence is greatly reduced (Westwater *et al*., 2007). The virulence phenotypes in *C. albicans* may therefore be linked to changes in immunogenicity of the *efg1* deletion caused by alterations in the cell wall (Sohn *et al*., 2003; Doedt *et al*., 2004; Zavrel *et al*., 2012). Deleting *EFG1* in *C. parapsilosis* also affects cell wall sensitivity (Fig. 3), and some of the genes differentially expressed in the *efg1* deletion (such as orthologues of *WSC2, WSC4*, *PGA26*, *PGA30, PGA62, ALS6, ALS7*) are known cell wall proteins in *C. albicans* (Table S5). The role of Efg1 in virulence is therefore likely to be conserved in both species.

There is a substantial overlap between the intergenic regions bound by Efg1 in *C. albicans* and *C. parapsilosis*, even when the *C. albicans* experiments were carried out using biofilm cells (Table S7; Nobile *et al*., 2012). In both species the targets are enriched for transcription factors (Table S3) and 38 of the 93 bound by Efg1 in *C. parapsilosis* are also bound in *C. albicans* biofilms (Table S7). These include Wor1, Wor2 and Czf1, which together with Efg1 control white-opaque switching in *C. albicans* (Zordan *et al*., 2007) (Figs 5 and 7). Most of the biofilm regulatory network identified by Nobile *et al*. (2012) is present (Efg1, Bcr1, Ndt80, Brg1, Tec1) (Fig. 7, Table S3). Other targets of Efg1 (e.g. Nrg1) that are associated with the yeast-to-hyphal switch (Lassak *et al*., 2011) are also bound by Efg1 in *C. parapsilosis* (Fig. 7). Efg1 and Ndt80 motifs co-occur in many intergenic regions in *C. parapsilosis* (Table S4). This strongly suggests that the components and structure of regulatory networks are highly conserved in *C. albicans* and *C. parapsilosis*. However, the functions of the networks are not as well conserved. For example, some of the phenotypes regulated by Efg1 in *C. albicans* (such as true hyphal growth and white/opaque switching) do not occur in *C. parapsilosis*. In addition, there is relatively little overlap between genes differentially expressed in an *efg1* deletion in *C. parapsilosis* and *C. albicans* (Table S5D) (Doedt *et al*., 2004). Even in biofilm development, where Efg1 is implicated in both species, many of the targets of the transcription factor are newly evolved in *C. albicans* but not in *C. parapsilosis* (Table S3C). It is therefore likely that the role of Efg1 in *C. parapsilosis* more closely resembles the ancestral state, and may have been adapted for filamentation, white-opaque switching and some aspects of biofilm regulation in *C. albicans*.

The intergenic regions bound by Efg1 in *C. parapsilosis* are significantly longer than the average intergenic region (Fig. 6B). It has also been reported that targets of Efg1 and other biofilm regulators (Nobile *et al*., 2012), and of Wor1 (Zordan *et al*., 2007), are longer in *C. albicans*. Nobile *et al*. (2012) suggest that this is partly because the biofilm targets are relatively young in evolutionary terms, and young genes tend to have long promoters. However, the association of promoter size with evolutionary age comes from an analysis of yeast species from before and after the whole-genome duplication (WGD) event. New promoters tend to be longer in post WGD species, but this is due to loss of genes within intergenic regions that occurs as part of a major rearrangement following duplication (Sugino and Innan, 2012). *Candida* species have not undergone genome duplication. The long promoters may be a feature of the number of regulatory proteins that bind to them; Efg1 appears to be part of many networks, and many targets are co-regulated. In *C. parapsilosis*, a significant number of the Efg1 targets are likely to also bind Ndt80, and possibly up to five other transcription factors. In addition, Efg1 binding, although centred on a short motif, extends over large regions of the promoters (Fig. 5). Finally, some of the transcription factors have very long 5′ untranslated regions; for *EFG1* in *C. albicans*, the 5′UTR extend almost 1200 bp upstream of the start (Srikantha *et al*., 2000). Some regulation may therefore be at the post-translational level.

Efg1 has many roles in *C. albicans*, including controlling filamentation (Stoldt *et al*., 1997), hypoxic response (Stichternoth and Ernst, 2009), regulation of cell wall proteins (Sohn *et al*., 2003; Zavrel *et al*., 2012), white-opaque switch (Morschhauser, 2010), biofilm development (Ramage *et al*., 2002; Nobile *et al*., 2012), generation of chlamydospores (Sonneborn *et al*., 1999a), regulation of metabolism (Doedt *et al*., 2004) and virulence (Lo *et al*., 1997; Chamilos *et al*., 2006; Pukkila-Worley *et al*., 2009). Some of these phenotypes (such as biofilm development, virulence and sensitivity to cell wall-damaging agents) are conserved in *C. parapsilosis* (Figs 2, 3 and 5; Zavrel *et al*., 2012). However, there are also significant differences. The role of Efg1 in biofilm development in *C. parapsilosis* is not as dominant as in *C. albicans*. More importantly, Efg1 circuits that regulate morphological switching in *C. parapsilosis* may have been adapted to control white-opaque switching and filamentation in *C. albicans*. We look forward to identifying the other members of the network regulating concentric-smooth switching, and to explore the roles of Efg1 and its targets in multiple cellular processes in *C. parapsilosis*.

## Experimental procedures

### Strains and growth conditions

*Candida parapsilosis* strains were grown in YPD medium (1% yeast extract, 2% peptone, 2% glucose) at 30°C. For colony selection 2% agar was added. To select for transformants, nourseothricin (Werner Bioagents Jena, Germany) was added to YPD agar at a final concentration of 200 μg ml^−1^. Transformants containing the *LEU2* and *HIS1* markers were selected on synthetic complete (SC; 0.67% yeast nitrogen base, 2% dextrose, 0.075% mixture of amino acids, 2% agar) media without leucine or histidine. For biofilm formation, *C. parapsilosis* was grown in synthetic defined (SD) medium (0.67% yeast nitrogen base) containing 50 mM glucose. Calcofluor white M2R (Sigma-Aldrich), caspofungin (from Cancidas, Merck & Co.) and Sodium n-Dodecyl Sulphate (SDS) (Biosciences) were added to the medium at the desired concentrations. For phenotype screening 500 μl of an overnight culture was washed and resuspended in phospho-buffered (PBS) at a concentration of 6.25 × 10^5^ cells ml^−1^. A fivefold serial dilution was generated and 3 μl was plated onto selected agar plates, and incubated at 30°C for 3 days. The strains and oligonucleotides used are listed in Table S8 and Table S9 respectively. All hypoxia experiments were carried out at 37°C in 1% O_2_ in an InVivo_2_ 400 hypoxia chamber. For microscopy, cells were taken straight from colonies on an agar plate and resuspended in 10 μl of 2 mM Calcofluor White. Images were taken using a Zeiss AxioImager M1 fluorescent microscope. All gene constructs are described in Fig. S1.

### Determination of switching rate

Colonies of *C. parapsilosis* strains with specific morphologies were isolated and grown overnight in 5 ml YPD at 30°C, shaking at 200 r.p.m. One millilitre of overnight cultures was washed twice in PBS and diluted to ∼ 100 cells per 100 μl in PBS. One hundred microlitres of the diluted sample was spread on a YPD plate. YPD plates were incubated under various temperatures and under normoxic and hypoxic conditions. Multiple plates were used so that > 1000 colonies were counted from each phenotype under each growth condition. The plates were inspected after 2 days, 3 days and 4 days. Colonies were counted on day three to determine switching frequencies (Table S1).

### Biofilm assays

Overnight cultures of *C. parapsilosis* strains grown in 5 ml YPD at 30°C were washed twice in 1 ml PBS buffer and diluted to an *A*_600_ of 1 in SD medium with 50 mM glucose. One millilitre of each culture was added to three wells of a 24-well Nunc Delta plate (Thermo Scientific) and incubated on a shaker (50 r.p.m.) at 37°C for 2 h (initial adhesion phase). The culture was removed and each well was washed once with 1 ml PBS buffer. One millilitre of fresh SD medium with 50 mM glucose was added to each well and the plates were shaken at 37°C for an additional 48 h. The culture was removed and wells were washed twice with 1 ml PBS buffer and allowed to dry at room temperature overnight. Biofilms were stained for 30 min with 500 μl of 0.4% (w/v) crystal violet. Crystal violet was removed and biofilms were washed twice with 1 ml PBS buffer and dried overnight at room temperature. The resulting biofilms were then photographed. For dry weight measurements of biofilms 5 ml of cultures at an *A*_600_ of 1 in SD medium with 50 mM glucose were inoculated in each well of six-well Nunc plates. Biofilms were allowed develop as above. After 48 h the wells were washed twice with 2 ml PBS buffer. One millilitre PBS buffer was added to each well and biofilm was scraped from the bottom of each well. Biofilm from two wells was combined, filtered using a 0.8 μm Millipore filter, dried for 48 h at room temperature and weighed. *In vivo* biofilms assays were carried out as described previously (Ding *et al*., 2011).

### Host damage assay

Human peripheral blood mononuclear cells (PBMCs) were isolated from buffy coats of healthy donors by Ficoll Paque PLUS (GE Healthcare) density gradient centrifugation, as described elsewhere (Schlenke *et al*., 1998). Experiments were performed according to the institutional regulation of the independent ethics committee of the University of Szeged. Isolated PBMCs were suspended in RPMI medium (LONZA) supplemented with 1% 100× Penicillin-Streptomycin solution (Sigma-Aldrich), and plated in 96-well cell culture plates (5 × 10^5^ PBMC per well, in 100 μl) to isolate monocytes by plastic adherence. After 2 h of incubation (37°C, 5% CO_2_, 100% relative humidity), floating cells were removed, and the attached monocytes were gently washed with PBS. The isolated cells were cultured for 7 days in X-VIVO 15 serum-free medium (LONZA) supplemented with 1% 100× Penicillin-Streptomycin solution (Sigma-Aldrich), in the presence of 10 ng ml^−1^ human recombinant GM-CSF (Sigma-Aldrich) to enable macrophage differentiation. Host-cell damaging capacity of *C. parapsilosis* strains was determined by LDH (lactate dehydrogenase) activity assay. PBMC-DM (PBMC-derived macrophage) cells were co-cultured with *C. parapsilosis* cells at a ratio of 1:5 in X-VIVO 15 serum-free medium (LONZA) supplemented with 1% 100× Penicillin-Streptomycin solution for 24 or 48 h, or left unstimulated (negative control). Macrophage cells alone incubated under identical conditions were applied as negative controls. LDH in medium from cultures containing uninfected and infected tissues at 24 and 48 h was measured using the Cytotoxicity Detection Kit (LDH; Roche) according to the manufacturer's instructions. Relative LDH activity was calculated, as described previously (Gacser *et al*., 2007).

### Galleria mellonella survival assay

Infection of *G. mellonella* larvae (Mous Livebait R.J., the Netherlands) was performed as described previously (Mylonakis, 2008; Garcia-Rodas *et al*., 2011). Briefly, larvae were inoculated with 10 μl of a *C. parapsilosis* suspension prepared in PBS containing 2 × 10^6^ yeast cells by an injection in the last left pro-leg, using a 26-gauge needle with Hamilton syringes. After injection, caterpillars were incubated at 25°C, and the number of dead larvae was noted daily. In each experiment, a group of caterpillars were left without any manipulation (untreated control), and another group of caterpillars was inoculated with PBS only.

### ChIP-seq and ChIP-PCR analysis

To introduce a tag just before the stop codon of Efg1, the MYC epitope tag and *C. albicans HIS1* gene were amplified by PCR from plasmid pFA-MYC-CaHIS1 (Lavoie *et al*., 2008) using primers EFG1_tag_F and EFG1_tag_R (Table S9). The purified product was transformed by electroporation into *C. parapsilosis* LCPH3 and LCPH4 (*EFG1/efg1::LEU2*, *his1−/his1−*) generating strains LCP3M and LCP4M. Correct integration of the tag was confirmed by PCR screening (Fig. S1).

Cultures were prepared for chromatin immunoprecipitation as described by Aparicio *et al*. (2005), from *C. parapsilosis* cells grown in YPD to an *A*_600_ = 1. Samples were sonicated using a Biorupter™ (Diagenode) for 15 min at 30 s intervals at high power setting in an ice bath. The ice was then changed and the sonication was repeated. Input samples before the addition of antibody were used as control. Regions bound to Efg1 were immunoprecipitated using a mouse monoclonal antibody (Myc-tag 9B11, Cell Signaling). Three input (before immunoprecipitation) and three immunoprecipitated samples were used (Table S10).

All ChIP-seq libraries were generated according to the Illumina protocol (Illumina Guide Part # 11257047 Rev. A). A limited 18-cycle amplification of size-selected libraries was carried out. To eliminate adapter dimers libraries were further sized selected using a 2.5% TAE agarose gels. Purified libraries were quantified using a Qubit™ fluorometer (Invitrogen) and a Quant-iT™ double-stranded DNA High-Sensitivity Assay Kit (Invitrogen). Clustering and sequencing of the material was carried out as per manufacturer's instructions on the Illumina GAIIx platform in the UCD Conway Institute. On average, 22.5 million 42 bp reads were obtained from each sample (Table S10).

The reads from the three experimental samples and the three controls were mapped to the latest version *C. parapsilosis* genome (Guida *et al*., 2011) using BWA (Li and Durbin, 2009). More than 90% of reads for each library were aligned to the reference (Table S10). Approximately 6.5% of reads from the immunoprecipitated samples and 1.2% of reads from the controls mapped to the rDNA locus. The Binary Alignment/Map (BAM) files were sorted and indexed using SAMtools (Li *et al*., 2009). Binding sites were predicted using MACS 2.0 (Zhang *et al*., 2008) (Feng *et al*., 2012), with a q-value threshold of 0.01 and automatic estimation of reads per genomic location. Fragment size was estimated using the SPP package (Kharchenko *et al*., 2008). Each immunoprecipitated sample was individually compared with the three merged controls. The reproducibility of the data sets was determined by pairwise comparisons of the three samples using IDR (Irreproducible Discovery Rate method), as recommended by the Encode consortium (Landt *et al*., 2012) and implemented in the SPP package (Kharchenko *et al*., 2008). A maximum of 1246 replicable peaks were identified. The MACS2 analysis was then repeated using the merged samples as input and called peaks were ranked by *P* value, using a False Discovery Rate (FDR) threshold of 1%. The top 1246 peaks were selected.

A custom Python pipeline was used to associate peaks with genes in the latest *C. parapsilosis* annotation (Guida *et al*., 2011). Peaks were mapped to the gene with the nearest 5′ end. Peaks lying in a divergent promoter region were assigned to both genes. Peaks lying between two convergent genes were not assigned. Peaks completely within a coding region were also not assigned. In total, 1155 peaks were assigned to 931 intergenic regions. Overall, these map to 536 unique promoters and 395 divergent promoters. Peaks were ranked by π value (Xiao *et al*., 2012), i.e. a product of *P*-values and fold enrichment at peak summit position with respect to the control. Where there were multiple peaks in a promoter, only the peak with the highest rank was considered (Table S3).

To confirm the peaks identified by ChIP-seq analysis, quantitative real-time PCR was carried out using three independent input and three immunoprecipitated samples from strains LCP3M and LCP4M. Primers were designed to amplify regions of high and low enrichment of Efg1 identified in the ChIP-seq experiments using Primer3Plus software (http://www.bioinformatics.nl/cgi-bin/primer3plus/primer3plus.cgi) (Table S9). For each reaction, a mastermix consisting of 5 μl 2× Brilliant III SYBR® green (Applied Biosystems), 500 nM of each primer, 0.15 μl ROX reference dye (diluted 1:500), 2.35 μl Nuclease free H_2_O and 1 μl of DNA (Input or Immunoprecipitated sample) was used. Samples were analysed in duplicate on a Strategene Mx3005P QPCR system (Agilent Technologies) using cycling conditions of one cycle at 95°C for 3 min followed by 40 cycles at 95°C for 10 s, 60°C for 20 s and a final cycle of 95°C for 1 min followed by melting curve analysis at 55°C to 95°C (temperature transition, 0.2°C s^−1^) with stepwise fluorescence detection. Enrichment in the promoter regions was calculated as the percentage of PCR product from the average of the immunoprecipitated samples normalized to the average of the input samples.

Motif prediction was carried out using the online version of DREME (Bailey, 2011) using 100 bp sequences around the summits of the best peak for each intergenic region for all samples. Shuffled input sequences were used for background comparison. Significant results were submitted to TOMTOM (Gupta *et al*., 2007) using default parameters. Motif occurrence was confirmed using FIMO with a significance threshold of 1e-03 (Grant *et al*., 2011).

### RNA-seq analysis

RNA-seq library preparation for strand-specific sequencing was carried out essentially as described in Guida *et al*. (2011), from cultures grown in YPD. We used HTSeq and DESeq (Anders and Huber, 2010) to compare 17.7 × 10^6^ 36 bp Illumina reads from three samples of wild type (*C. parapsilosis* CLIB214) to 15 × 10^6^ reads from two different *efg1* deletion strains in the smooth phenotype and 16.4 × 10^6^ reads from three deletions of the concentric phenotype (Table S10). Reads with exceptionally high coverage (rDNA) were masked. The RNA-seq data sets were submitted to the Gene Expression Omnibus (GEO) (series GSE41065). All Gene Ontology analysis was carried out at the Candida Genome Database (Inglis *et al*., 2012).

### Gene age analysis

A total of 585 genes present in the *C. albicans, C. dubliniensis* and *C. tropicalis* and not in other CTG clade species were designated as ‘young’ on the *C. albicans* branch, whereas 473 genes present only in the *C. parapsilosis, C. orthopsilosis* and *L. elongisporus* branch were designated ‘young’ in *C. parapsilosis*. Gene orthology was defined using the Candida Gene Order Browser (version 29 August 2012) (Fitzpatrick *et al*., 2010; Maguire *et al*., 2013). Differences between observed and expected values in the list of Efg1-bound genes were determined using Fisher's exact test (Table S3C).
