# Supplementary material for: The APSES transcription factor Efg1 is a global regulator that controls morphogenesis and biofilm formation in Candida parapsilosis
Source: Mol Microbiol. 2013 Aug 15;90(1):36–53. doi: 10.1111/mmi.12345 (PMC3912905; doi:10.1111/mmi.12345)
Supplement: Supplementary file 1 [file mmi0090-0036-sd1.pdf]

Table S1. Switching frequencies in *C. parapsilosis* strains.

Strain Name	Description	Concentric to Smooth (%) Average (range)	Smooth to concentric (%) Average (range)	Sectored colonies (%)
LCP1	*efg1::FRT/ efg1::FRT*, using SAT1 flipper	28.8 (18.1-31.6)	14.1 (12.6-17.2)	
LCP2	*efg1::FRT/ efg1::FRT*, using SAT1 flipper	26.6 (22.4-30.3)	11.4 (9.0-15.2)	
LCP3	*leu2::FRT/leu2::FRT, his1::FRT/his1::FRT, efg1::LEU2/ efg1::HIS1,* using long primer method	25.8 (18.3-32.1)	20.6 (14.1-25.3)	
LCP4	*leu2::FRT/leu2::FRT, his1::FRT/his1::FRT, efg1::LEU2/ efg1::HIS1,* using long primer method	18.5 (15.3-23.2)	19.7 (15.0-24.2)	
LCP5	*leu2::FRT/leu2::FRT, his1::FRT/his1::FRT, efg1::LEU2/ efg1::HIS1,* using Fusion PCR method	11.6 (8.5-14.2)	27 (23.7-31.5)	
LCP6	*leu2::FRT/leu2::FRT, his1::FRT/his1::FRT, efg1::LEU2/ efg1::HIS1,* using Fusion PCR method	4.8 (2.0-8.3)	33.6 (23.8-31.7)	
LCP7	*leu2::FRT/leu2::FRT, his1::FRT/his1::FRT, efg1::LEU2/ efg1::HIS1,* using Fusion PCR method	65.1 (61.9-71.4)	7.4 (4.0-11.2)	2.2 (1.1-2.5)
LCP8	*leu2::FRT/leu2::FRT, his1::FRT/his1::FRT, efg1::LEU2/ efg1::HIS1,* using Fusion PCR method	81 (71.8-89.5)	3.9 (2.3-5.0)	2.6-5.1
LCP1E	*efg1::FRT/ efg1::FRT, efh1::FRT/efh1::FRT* using *SAT1* flipper	26.3 (21.2-31)	12.5 (9.8-16.7)	
LCP7_RI	*leu2::FRT/leu2::FRT, his1::FRT/his1::FRT, efg1::LEU2/ efg1::HIS1, pACT1-EFG1:SAT*	0.4(0.3-0.5)	1.6 (1.0-2.4)	9.7(8.6-10.7)
CLIB214	*leu2::FRT/leu2::FRT, his1::FRT/his1::FRT, efg1::LEU2/ efg1::HIS1,* using Fusion PCR method	0.2 (0.1-0.4)	1.9 (1.4-2.5)	8.3 (7.5-9.7)

The switching rates between a concentric phenotype and a novel crater-like pheonotype observed in the *efh1* mutant strains.

Strain Name	Description	Concentric to crater	Crater to Wrinkled	Sectoring
LHP93	*leu2::FRT/leu2::FRT, his1::FRT/his1::FRT, efh1::LEU2/efh1::HIS1,* using Fusion PCR method	2.4 (0.8-4.4)	2.3 (2.1-2.5)	0.2 (0-0.5)
LHP95	*leu2::FRT/leu2::FRT, his1::FRT/his1::FRT, efh1::LEU2/efh1::HIS1,* using Fusion PCR method	0.7 (0.4-0.9)	3.2 (2.3-4.3)	

Table S2. Cell morphology in colonies during growth in normoxic and hypoxic conditions.

	Hypoxic (1% O_2)<br><br>% round yeast cells	Hypoxic (1% O_2)<br><br>% elongated<br><br>/pseudohyphal cells	Normoxia (21% O_2)<br><br>% round yeast cells	Normoxia (21% O_2)<br><br>% elongated<br><br>/pseudohyphal cells
Wildtype (concentric)	82-87	13-18	30-34	66-70
Wildtype (smooth)	91-95	9-12	95-98	2-5
efg1::LEU2/efg1::HIS1<br><br>(LCP5, concentric)	8-14 *	86-92 *	35-40	60-65
efg1::LEU2/efg1::HIS1<br><br>(LCP5, smooth)	8-16 *	84-92 *	75-83 *	16-25 *
efg1::LEU2/efg1::HIS1<br><br>(LCP7, concentric)	7-12 *	88-93 *	30-41	59-70
efg1::LEU2/efg1::HIS1<br><br>(LCP7, smooth)	10-17 *	83-90 *	77-88 *	12-23 *
efg1::LEU2/efg1::HIS1/ACT1-EFG1<br><br>(Concentric)	68-80	20-32 *	36-39	61-64
efg1::LEU2/efg1::HIS1/ACT1-EFG1<br><br>(Smooth)	79-86	14-21	81-84 *	16-19 *
efg1::FRT/efg1::FRT/ efh1::FRT/efh1::FRT<br><br>(Concentric)	5-16 *<br><br>(P = 0.8)^	84-95 *<br><br>(P=0.9)^	29-46<br><br>(P=0.7) ^	54-71<br><br>(P=0.8)^
efg1::FRT/efg1::FRT/ efh1::FRT/efh1::FRT<br><br>(Smooth)	4-18 *<br><br>(P=0.9)^	82-96 *<br><br>(P=0.8)^	69-82 *<br><br>(P=0.5) ^	18-31 *<br><br>(P=6)^

Cells from colonies were stained with calcofluor white and examined microscopically. 1000 cells from each strain were counted, and the percentages of the indicated cell types were calculated. This was repeated 3 times and the ranges are shown.

*indicates significant changes (P-value < 0.05) from wild type.

^Differences between *efg1/efh1* double deletion and *efg1* single deletion are not significant.

Tables S3-S7 included as searchable Excel files.

Table S8 list of strains used.

Species	Strain	Origin	Genotype
C. parapsilosis	CLIB214	Type strain	
C. parapsilosis	CPL2H1	Derived from CLIB214 (Holland et al, in prep)	*leu2::FRT/leu2::FRT, his1::FRT/his1::FRT*
C. parapsilosis	CPRI	Derived from CLIBL2H1 (Holland et al, in prep)	*leu2::FRT/leu2::FRT, his1::FRT/his1::FRT, frt::CmLEU2/frt::CdHIS1*
C. parapsilosis	LCP1H	Derived from CLIB214 using *SAT1* flipper cassette method.	*efg1::FRT/EFG1*
C. parapsilosis	LCP1	Derived from CLIB214 using *SAT1* flipper cassette method.	*efg1::FRT/ efg1::FRT*
C. parapsilosis	LCP2	Derived from CLIB214, using *SAT1* flipper cassette method.	*efg1::FRT/ efg1::FRT*
C. parapsilosis	LCPH3	Derived from CLIBL2H1, using long primer method.	*leu2::FRT/leu2::FRT, his1::FRT/his1::FRT, efg1::LEU2/EFG1*
C. parapsilosis	LCPH4	Derived from CLIBL2H1, using long primer method.	*leu2::FRT/leu2::FRT, his1::FRT/his1::FRT, efg1::LEU2/EFG1*
C. parapsilosis	LCP3	Derived from CLIBL2H1, using long primer method.	*leu2::FRT/leu2::FRT, his1::FRT/his1::FRT, efg1::LEU2/ efg1::HIS1*
C. parapsilosis	LCP4	Derived from CLIBL2H1, using long primer method.	*leu2::FRT/leu2::FRT, his1::FRT/his1::FRT, efg1::LEU2/ efg1::HIS1*
C. parapsilosis	LCP5	Derived from CLIBL2H1, using Fusion method	*leu2::FRT/leu2::FRT, his1::FRT/his1::FRT, efg1::LEU2/ efg1::HIS1*
C. parapsilosis	LCP6	Derived from CLIBL2H1, using Fusion method	*leu2::FRT/leu2::FRT, his1::FRT/his1::FRT, efg1::LEU2/ efg1::HIS1*
C. parapsiloiss	LCP7	Derived from CLIBL2H1, using Fusion method	*leu2::FRT/leu2::FRT, his1::FRT/his1::FRT, efg1::LEU2/ efg1::HIS1*
C. parapsilosis	LCP8	Derived from CLIBL2H1, using Fusion method	*leu2::FRT/leu2::FRT, his1::FRT/his1::FRT, efg1::LEU2/ efg1::HIS1*
C. parapsilosis	LHP93	Derived from CLIBL2H1, using Fusion method	*leu2::FRT/leu2::FRT, his1::FRT/his1::FRT, efh1::LEU2/efh1::HIS1*
C. parapsilosis	LHP95	Derived from CLIBL2H1, using Fusion method	*leu2::FRT/leu2::FRT, his1::FRT/his1::FRT, efh1::LEU2/efh1::HIS1*

C. parapsilosis	LCP1E	Deriverd from LCP1	*efg1::FRT/ efg1::FRT/ efh1::FRT/efh1::FRT*
C. parapsilosis	LCP3M	Derived from LCPH3	*leu2::FRT/leu2::FRT, his1::FRT/his1::FRT, efg1::LEU2/EFG1-MYC-HIS1*
C. parapsilosis	LCP4M	Derived from LCPH4	*leu2::FRT/leu2::FRT, his1::FRT/his1::FRT, efg1::LEU2/EFG1-MYC-HIS1*

Table S9. List of oligonucleotide primers used.

Primers	Sequence
EFG1_1f	5'-GGGGGGTACCTTAAACGCCGACTGGAAATA-3’
EFG1_2r	5'-GGGGGGGCCCCTGTGGAGCACTTGCACTGT-3’
EFG1_3f	5'-GGGGCCGCGGGCAGCTGCCAATTCAGAAC-3'
EFG1_4r	5'-GGGGGAGCTCAATGCACGTACCCCAGACAT -3’
But237	5'- GCTGTTCCGTTATGTGTAATCATCC 3'
EFG1_5f	5'- TTGAAGTCCAATCATCGGTTT -3'
EFG1_6r	5'- CCCGGGTACTGGTAACGACT -3'
EFG1_7r	5'- CGTAATGCGATTGTTGGTTG -3'
EFG1_80auxoF	5’ATGTCTACTTATTCATTGCCACTTAGATCTCAAATGAACGGAAACTACAACAGGCAAGTGCTCCACAGAGCGGAGCAGCACCAGTGTGATGGATATCTGC-3’
EFG1_80auxoR	5’TTACTTTCCAACTTTTGGCAGATGCTGCGTATGCTGATGTGGTGATACATGTACTCCTCCAACACTAGCTGATGGTTGCTAGCTCGGATCCACTAGTAACG-3’
LeuCheck1	5’-GAAGTTGGTGACGCGATTGT-3’
LeuCheck2	5’-TTCCCCTTCAATGTATGCAA-3’
HisCheck1	5’-AAAATCAATGGGCATTCTCG-3’
HisCheck2	5’-TGGGAAGCAGACATTCAACA-3’
Univeralko2	5’CCGCTGCTAGGCGCGCCGTGACCAGTGTGATGGATATCTGC-3’
EFG1-1	5’-TTAAACGCCGACTGGAAATA-3’
EFG1-3	5’CACGGCGCGCCTAGCAGCGGGAATTTAAATACAGGTAGTTG-3’
EFG1-4	5’GTCAGCGGCCGCATCCCTGCAGAGGTGCCATCGAAAGAAG-3’
EFG1-5	5’GCAGGGATGCGGCCGCTGACACATAACTTTCCAGAAGCGCAGCTCGGATCCACTAGTAACG-3’
EFG1-6	5’-AGTGCCCGATGAAACAGAAC-3’
EFG1-5’Check	5’-CAAACAAGACAAACCGTCCTG-3’
EFG1-3’Check	5’-GCAGAGCGAAAGAGCAAAGA-3’
EFH1-1	5’-TGGTCGGTATCCTTTTCGAG-3’
EFH1-3	5’CACGGCGCGCCTAGCAGCGGTTATTCACGTGTGTAGTGAT-3’
EFH1-4	5’GTCAGCGGCCGCATCCCTGCTAAGGCAGTTGATTTTGGCTAA-3’
EFH1-5	5’GCAGGGATGCGGCCGCTGACAAGTAGACTGAACTCCGCGCAGCTCGGATCCACTAGTAACG-3’

EFH1-6	5’-TGGCTCACGATGAAAATCAG-3’
EFH1-5’Check	5’-AAACTGGGCCGAAAAAGATT-3’
EFH1-3’Check	5’-ATTGGAAAAGTCGGTCGTTG-3’
EFG1_tag_f	5’AGCAACCATCAGCTAGTGTTGGAGGAGTACATGTATCACCACATC AGCATACGCAGCATCTGCCAAAAGTTGGAAAGGAAGCTTCGTACG CTGCAGGTC-3’
EFG1_tag_r	5’TTACCCTCAGCTATGTTCACGATGTGAGCCACTGCTACTCCCCTTC TGCTTCTTCTTTCGATGGCACCTCTTTATCTGATATCATCGATGAAT TCGAG-3’
CpAct1(-1000)	5’-GGAGCGGGGCCCCACAGCCTGTGTAATAAATCTT-3’
CpAct1(0)	5’-TGTAGTGACAAATAATCAAAGGTTC-3’
ACT1_Check	5’-CGCTCTAGACCCAATAATCACC-3’
EFG1_xho_f	5’**GAACCTTTGATTATTTGTCACTACA**TTCATGTCTACTTATTCATT GCCAC-3’
EFG1_xho_r	5’-GGCGCCGTCGA**G**GCCACCAAACCACTATAAATA-3’
Efh1_1.1	5’- **GGGG GGTACC** TGGTCGGTATCCTTTTCGAG-3’
Efh1_2.1	5’- **GGGG GGGCCC** TTATTCACGTGTGTAGTGAT-3’
Efh1_3.1	5’- **GGGG CCGCGG** TAAGGCAGTTGATTTTGGCTAA-3’
Efh1_4.1	5’- **GGGG GAGCTC** TGGCTCACGATGAAAATCAG-3’

Primers for qRT-PCR	Target Gene	Sequence
ADAEC_rt_f	CPAR2_211420	5’-ACCACCACTCAATGATGCAG-3’
ADAEC_rt_r	CPAR2_211420	5’-CAACCACCTTCTTCCTCACC-3’
AOX1_rt_f	CPAR2_700950	5’-AGCTGATTGCTGGTGTTCCT-3’
AOX1_rt_r	CPAR2_700950	5’-TGCAAAGTCTCAATCCATGC-3’
FCR1_rt_f	CPAR2_808620	5’-TGCAAATGATCAAGCGTTGT-3’
FCR1_rt_r	CPAR2_808620	5’-GCTGTTGCTGTTTGTGCTGT-3’
GAD1_rt_f	CPAR2_804600	5’-TGGTGCTTTTGAACCAGTTG-3’
GAD1_rt_r	CPAR2_804600	5’-AAATGGAGCGACAAATCCAC-3’
PGA62_rt_f	CPAR2_403180	5’-TACACAACCTGGTGCCCATT-3’
PGA62_rt_r	CPAR2_403180	5’-CGACAGTGGTTGAGATTTCG-3’
UME6_rt_r	CPAR2_803820	5’-CATTCCCGGTGCTATTGTTT-3’
UME6_rt_f	CPAR2_803820	5’-CTTGGGCATCCTGTTTTGAT-3’

ChIP Confirm Primers	Target Promoter Region	
BCR1_chip_1f	CPAR2_205990	5’-ACTTTTAGGTTTGGAGCCATTG-3’
BCR1_chip_1r	CPAR2_205990	5’-CCAACAATAACAAATGCCACTC-3’
BCR1_chip_2f	CPAR2_205990	5’-TGGTGCAATTATAAACGACTGG-3’
BCR1_chip_2r	CPAR2_205990	5’-AGCCACGATCCTATGTTTTAGG-3’
BCR1_chip_3f	CPAR2_205990	5’-CTACCCGTTCTACAACCGTCTC-3’
BCR1_chip_3r	CPAR2_205990	5’-TCTGTCGTTTCGGTTAAATGC-3’
BRG1_chip_1f	CPAR2_107940	5’-CCCCAACTCTATTCAACAAACC-3’
BRG1_chip_1r	CPAR2_107940	5’-CTGTCGTGAAACTCGTTCACTC-3’
BRG1_chip_2f	CPAR2_107940	5’-AGTGGCATACACACTTCACTGG-3’
BRG1_chip_2r	CPAR2_107940	5’-TTGTGCGTTGTTTTTCATTTTG-3’
BRG1_chip_3f	CPAR2_107940	5’-AATATCCGTGTCTCTTGTGCAG-3’
BRG1_chip_3r	CPAR2_107940	5’-GAGGCTGCTGATTACACAAACC-3’
BRG1_chip_4f	CPAR2_107940	5’-CCGCTACTTCCACTGTTTCTTC-3’
BRG1_chip_4r	CPAR2_107940	5’-TGCAGTTGTTGTGAAAGTTGTG-3’
CZF1_chip_1f	CPAR2_501290	5’-AGGCTGTTTGCCACAGTATAGC-3’
CZF1_chip_1r	CPAR2_501290	5’-TTGATCACCCATGCATATCTTC-3’
CZF1_chip_2f	CPAR2_501290	5’-ACTGCATATGGGAATGACTGTG-3’
CZF1_chip_2r	CPAR2_501290	5’-CGCTGATGATACATTTTCCAAC-3’
CZF1_chip_3f	CPAR2_501290	5’-ATCCATATGGTAACCCAAGCAG-3’
CZF1_chip_3r	CPAR2_501290	5’-TTGGAGTTTTGTTGTTGGTACG-3’
NDT80_chip_1f	CPAR2_213640	5’-TTGGATAATGGTGAGTTTGCTG-3’
NDT80_chip_1r	CPAR2_213640	5’-GAGGTGTTCCTTCTGCTTATGG-3’
NDT80_chip_2f	CPAR2_213640	5’-TGCATGCAGTACAATGCTCTC-3’
NDT80_chip_2r	CPAR2_213640	5’-AGCCCCCAAGTCTAAAACAAC-3’
NDT80_chip_3f	CPAR2_213640	5’-AGTACCACAATCCCCATCAAAC-3’
NDT80_chip_3r	CPAR2_213640	5’-CTCTTTTGAAGCTCGCTTTACC-3’
NDT80_chip_4f	CPAR2_213640	5’-TAGGAGTCTCCCCTCTCAACTG-3’
NDT80_chip_4r	CPAR2_213640	5’-AGTTTTGCGAAAGAAGCAAGTC-3’

NRG1_chip_1f	CPAR2_300790	5'-TCGAGGAACTCGTTACATTTTG-3'
NRG1_chip_1r	CPAR2_300790	5'-TTCCTTCCCGCTATTTTGTAAC-3'
NRG1_chip_2f	CPAR2_300790	5'-CCCAAGGTGGATGAAAGTAAAC-3'
NRG1_chip_2r	CPAR2_300790	5'-ACGACGGGAAAAAGAATGTAAC-3'
NRG1_chip_3f	CPAR2_300790	5'-TCTCGCACAGACTTTACAAACG-3'
NRG1_chip_3r	CPAR2_300790	5'-CCAGAACGTCTTCATCAGTGAC-3'
NRG1_chip_4f	CPAR2_300790	5'-GGAGCAAAGTTGTGGGTTAAAG-3'
NRG1_chip_4r	CPAR2_300790	5'-CCAGCATTCAAAACACACTCAC-3'
WOR2_chip_1f	CPAR2_405400	5'-CTTACTTTGCGTATTGCTGCAC-3'
WOR2_chip_1r	CPAR2_405400	5'-TCATTATGGAGCAACACACTCC-3'
WOR2_chip_2f	CPAR2_405400	5'-ATGGCTAGAATTCTTGGTACGG-3'
WOR2_chip_2r	CPAR2_405400	5'-CAACACCAAGTGAGAGAAAACG-3'
WOR2_chip_3f	CPAR2_405400	5'-AGCTTTTCCACTCTCTCCACAG-3'
WOR2_chip_3r	CPAR2_405400	5'-GTCTCGGTTTAAATTGCCTACG-3'
WOR2_chip_4f	CPAR2_405400	5'-CGCCTATTCCACTTACACTTCC-3'
WOR2_chip_4r	CPAR2_405400	5'-CTTGACCTTATAGCCCCATCTG-3'

Table S10. Strains and libraries used for RNA-seq and ChIP-seq.

Experiment	Strain	Library	Total reads	Mapped reads %	rRNA reads %
ChIP-Seq	LCP3M	Sample 1	22.9M	83.5%	6.5%
	LCP3M	Control 1	21.5M	96.4%	1.2%
	LCP3M	Sample 2	20.7M	83.8%	7.4%
	LCP3M	Control 2	23M	97.6%	1.4%
	LCP4M	Sample 3	22.6M	81.7%	5.7%
	LCP4M	Control 3	24.3M	97.7%	0.8%
RNA-Seq	CLIB214	Wild type 1	5M	97.3%	2.7%
	CLIB214	Wild type 2	8.5M	97.6%	2.5%
	CLIB214	Wild type 3	4.6M	98.1%	0.1%
	LCP5	*efg1* smooth 1	7M	98.3%	3.8%
	LCP7	*efg1* smooth 2	8.1M	97.9%	1.6%
	LCP5	*efg1* concentric 1	5M	95.7%	2.8%
	LCP6	*efg1* concentric 2	7.7M	98.2%	3.0%
	LCP8	*efg1* concentric 3	4.2M	98.0%	1.7%

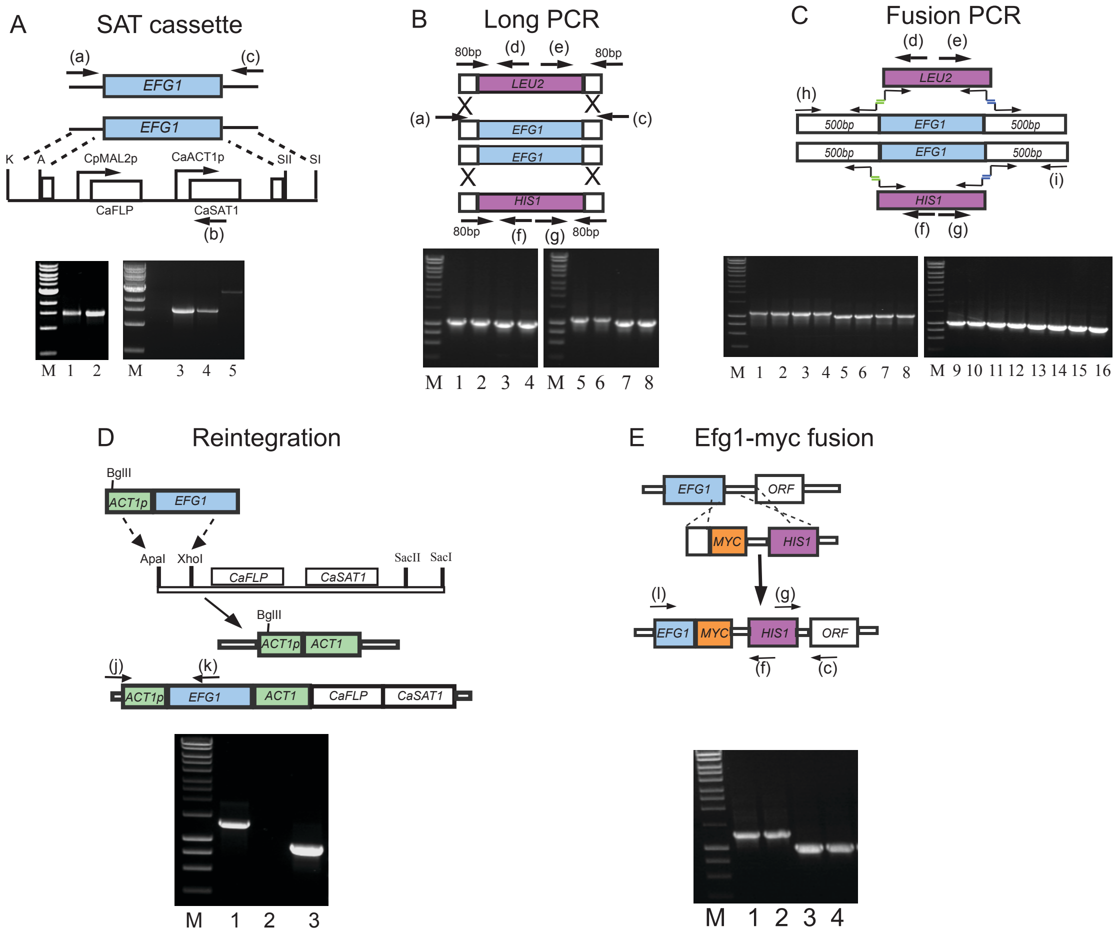

Fig S1. Efg1 constructions in *C. parapsilosis*.

The oligonucleotides used are listed in Table S9 and the strains are listed in Table S8.

A) Replacement of *EFG1* alleles with SAT-flipper.

Knockouts were generated in *C. parapsilosis* CLIB214 using the *SAT1* flipper as described in Ding and Butler (2007). A 542 bp fragment 5' to the *EFG1* ORF was amplified from *C. parapsilosis* CLIB214 genomic DNA using GoTaq DNA polymerase (Promega) using primers efg1_1f and efg1_2r (containing recognition sites for *Kpn*I and *Apa*I) and a 474 bp fragment downstream region using primers efg1_3f and efg1_4r (containing recognition sites for *Sac*II and *Sac*I) (Table S8). The PCR products were digested with the relevant enzymes and cloned into the flanking sites of pCD8 (Ding & Butler, 2007) generating plasmid LC1001. The entire cassette and *EFG1* flanking sequences were excised by digesting with *Kpn*I (*K*) and *Sac*I (*SI*), and transformed into *C. parapsilosis* CLIB214 by electroporation. The cassette was recycled by activating the Flp recombinase, and the second allele was disrupted using the same cassette, using methods described in Ding and Butler (Ding & Butler, 2007). Two independent strains (*C. parapsilosis* LCP1 and LCP2) were confirmed by PCR.

LCP1: lanes 1 and 3; LCP2: lanes 2 and 4; CLIB214: lane 5

Lane M: Molecular weight ladder (1 kb Ladder (New England BioLabs)); Lanes 1 and 2, amplification with primers EFG1_5f (a) and But237 (b) in LCP1 and LCP2 yielding a fragment of 1.5 kb. Lanes 3-5, amplification with primers EFG1_5f (a) and EFG1_7r (c) giving a fragment of 1.5 kb in LCP1 and LCP2 and 2.7 kb in wild type (lane 5).

B) Replacement of *EFG1* alleles with *LEU2* and *HIS1* by homologous recombination.

Gene knockouts were generated in *C. parapsilosis* CPL2H1 (*his1-, leu2-*, Holland et al, in prep) by amplifying the *LEU2* gene from *C. maltosa* and the *HIS1* gene from *C. dubliniensis* from plasmids pSN40 and pSN52 (Noble & Johnson, 2005) using primer Efg1_80auxoF, which contains 80bp complementary to the upstream flanking region of *EFG1* and primer Efg1_80auxoR which contains 80bp complementary to the downstream flanking region of *EFG1* (Table S8). The resulting PCR fragments were purified using a PCR Purification Kit (Qiagen) and transformed directly into *C. parapsilosis* CPL2H1 generating strains LCP3 and LCP4. Correct integration was confirmed by PCR.

Strain LCP3: lanes 1, 3, 5, 7; Strain LCP4: lanes 2, 4, 6, 8

Lane M: Molecular weight ladder, (Hyperladder I ™ (Bioline));

Lanes 1 and 2, amplification with primers EFG1_5f (a) and LeuCheck1 (d) giving a fragment of 1.1 kb, lanes 3 and 4, amplification with LeuCheck2 (e) and EFG1_7r (c), giving a fragment of 0.96 kb, lanes 5 and 6, amplification with primers EFG1_5f (a) and HisCheck1 (f), giving a fragment of 1.1 kb, lanes 7 and 8, amplification with primers HisCheck2 (g) and EFG1_7r (c), giving a fragment of 1.0 kb.

C) Replacement of *EFG1* alleles with *LEU2* and *HIS1* by fusion PCR.

The *LEU2* gene from *C. maltosa* and the *HIS1* gene from *C. dubliniensis* were amplified from plasmids pSN40 and pSN52 (Noble & Johnson, 2005), respectively, using primers Universalko2 and EFG1-5. 500 bp fragments were amplified from upstream of *EFG1* using primers EFG1-1 and EFG1-3, and from downstream using primers EFG1-4 and EFG1-6. The fusion primers have complementary ends shown in blue and green. The entire fragment (5' *EFG1-LEU2/HIS1-EFG1*-3') was amplified using primers EFG1-1 and EFG1-6, and transformed into strain CPL2H1. Four independent knockouts (LCP5-LCP8) were confirmed by PCR.

Strain LCP5: lanes 1, 5, 9, 13; Strain LCP6: lanes 2, 6, 10, 14; Strain LCP7: Lanes 3, 7, 11, 15; Strain LCP8: lanes 4, 8, 12, 16.

Lane M.: Molecular weight ladder (Hyperladder I (Bioline); Lanes 1-4 amplification with primers EFG1-5'check (a) and LeuCheck1 (d) giving a product of 1.1 kb, lanes 5-8, amplification with primers EFG1-3'Check (i) and LeuCheck2 (e), giving a fragment of 0.9 kb, lanes 9-12, amplification with primers EFG1-5'Check (h) and HisCheck1 (f), giving a fragment of 0.9 kb, lanes 12-16, amplification with primers HisCheck2 (g) and EFG1-3'Check (i), giving a fragment of 1.0 kb.

D) Re-integration of *EFG1*

An *ACT1-EFG1* fusion was generated by amplifying the *ACT1* promoter using primers CpAct1(1000) (containing an ApaI recognition site) and CpAct1(0) and the a 1.6 kb fragment of the *EFG1* gene using primers EFG1_xho_f (containing a sequence complementary to Cp Act1(0)) and EFG1_xho_r (containing an XhoI recognition site). The PCR products were joined together by fusion PCR, digested with *Apa*I and *Xho*I and cloned into the equivalent sites in the pSFS2A plasmid, which contains a *SAT1* cassette (Reuss *et al.*, 2004). The plasmid was linearised with *Bgl*II to direct integration to one allele of the *ACT1* locus, and was transformed into strain LCP7 (*efg1*/*efg1*) by electroporation. One nourseothricin-resistant (LCP7_RI) was chosen. Correct integration of the *EFG1* gene was determined by PCR.

Strain LCP7_RI: lanes 1 and 2; Wildtype (CLIB214); lane 3.

Lane M: Molecular weight ladder (Hyperladder I (Bioline). ; Lane 1, amplification of LCP7_RI DNA with primers CpAct1-check (j) and EFG1_3r (k) giving a product of 1.3 kb. Lane 2, Amplification of LCP7_RI DNA using primers EFG1_5f and EFG1_6r produces no fragment which confirms that the endogenous EFG1 gene is deleted. Lane 3 is a control lane, showing amplification of CLIB214 DNA with primers EFG1_5f and EFG1_6r giving a fragment of 949 bp.

E) Generation of an Efg1-myc fusion

A MYC tag was introduced at the C terminus of Efg1 generating strains LCP3M and LCP4M by homologous recombination. The bottom panel shows PCR confirmation of the integration in two independent constructions. Lane M: molecular weight marker (HyperLadder I from Bioline); lanes 1 and 2, amplification with primers EFG1_3f (l) and HisCheck1 (f) giving a fragment of 1.14 kb; lanes 3 and 4, amplification with primers HisCheck2 (g) and EFG1_7r (c) giving a fragment of 0.98 kb.

Figure S2.

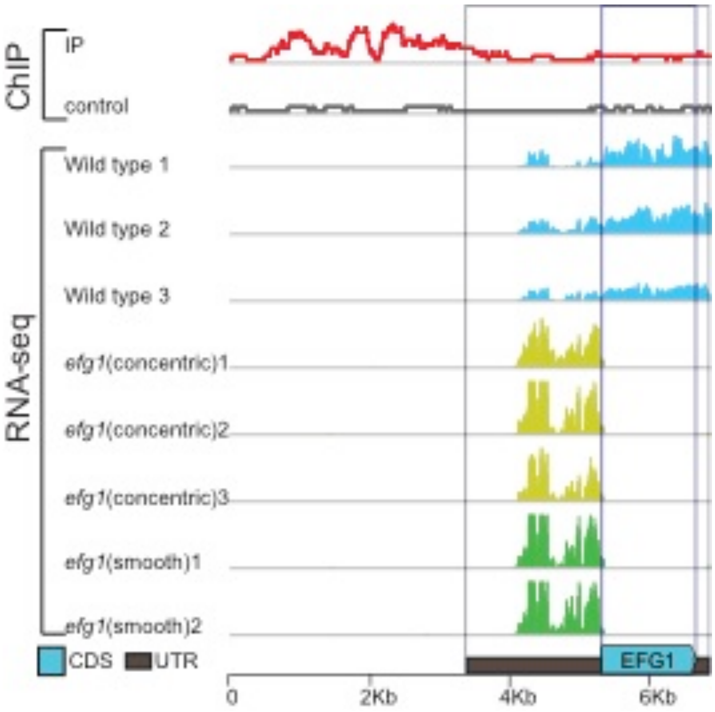

Figure S2. *EFG1* is autoregulated.

The diagram shows the region surrounding the *C. parapsilosis EFG1* Open Reading Frame (blue box) and the 5' and 3' UTR regions. The entire ORF (but not the UTR regions) were deleted in *efg1* strains. The ChIP tracks show binding of Efg1 to the upstream region of the gene (a large binding peak downstream of the gene is not shown). In wild type cells the entire *EFG1* region is transcribed, with the 5'UTR being transcribed at a low level. When the *EFG1* ORF is deleted, expression of the 5'UTR is greatly increased in both concentric and smooth cells, suggesting that Efg1 represses its own expression.

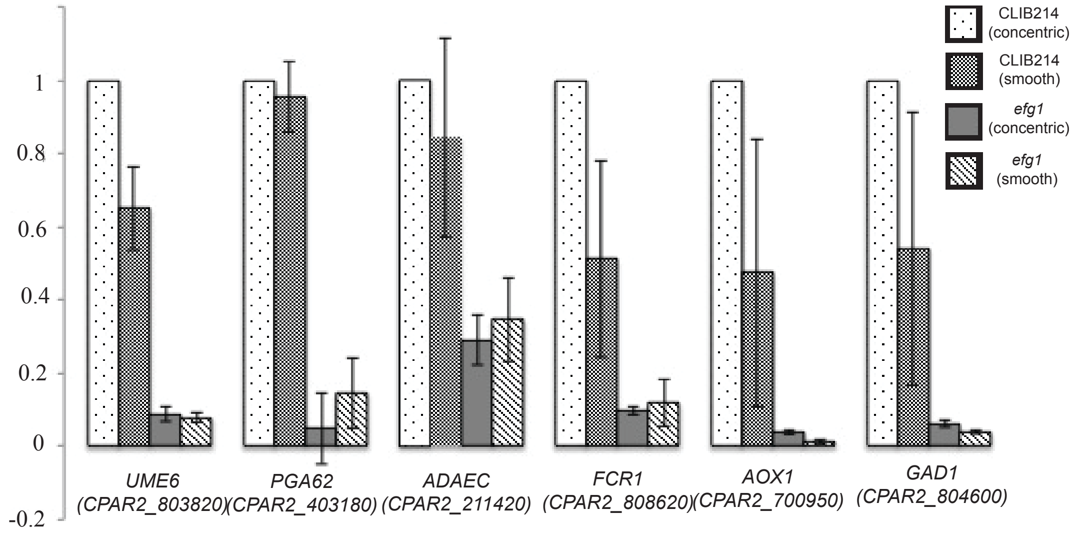

Figure S3. Confirmation of gene expression using quantitative RT-PCR.

Expression of several genes with decreased levels in an *efg1* deletion (RNA-seq) and bound by Efg1 (ChIP-seq) was confirmed by qRT-PCR.

cDNA from both smooth and concentric phenotypes of strains LCP1, LCP5, LCP7 and CLIB214 was used for expression analysis. Quantitative RT-PCR was carried out with Brilliant III SYBR green using the Strategene Mx3005P QPCR system (Agilent Technologies). Cycling conditions consisted of one cycle at 95°C for 3 min followed by 40 cycles of 95°C for 10 s, 60°C for 20 s and a final cycle of 95°C for 1 min followed by melting curve analysis at 55°C to 95 °C (temperature transition, 0.2°C/s) with stepwise fluorescence detection. All primers used for analysis are listed in Table S8. Expression was normalized to *ACT1* and is shown relative to expression of the wild type concentric phenotype. The average of three biological replicates and the standard deviation is shown.

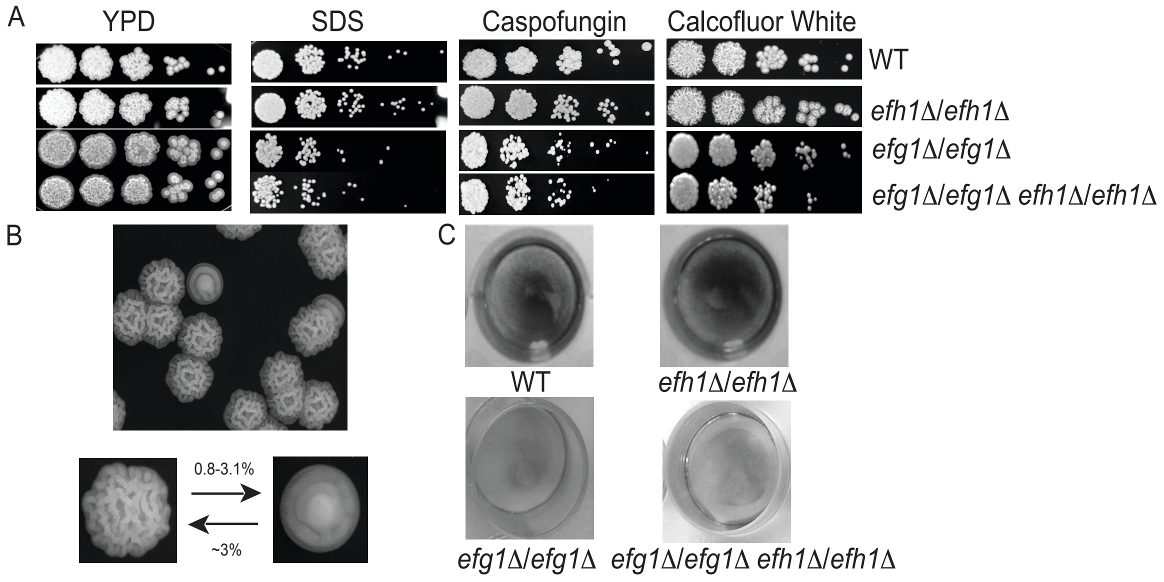

Figure S4: Deleting *EFH1* has little effect on *EFG1*-dependent phenotypes.

Both alleles of *EFH1* (CPAR2_104660) were deleted using the fusion PCR method in the wild type background (CPL2H1) and in an *efg1/efg1* mutant (LCP1) using the *SAT1* flipper method (See Fig. S1).

A) Deleting *EFH1* has no effect on the susceptibility to cell wall stresses, and the *efg1/efh1* double deletion has a similar phenotype to deleting *EFG1* alone.

B) Deleting *EFH1* has no effect on switching between concentric and smooth colonies. However, a new "crater" morphology is observed at a low frequency.

C). Deleting *EFH1* has no effect on biofilm development on Nunc plates, and the *efg1/efh1* double deletion has a similar phenotype to deleting *EFG1* alone.

WT= *C. parapsilosis* CLIB214, *efh1Δ/ efh1Δ*= *C. parapsilosis* LHP93, *efg1Δ/ efg1Δ* = LCP1, *efg1Δ/ efg1Δ efh1Δ/ efh1Δ* = LCP1E
